# Preparation, Characterization and In Vitro Biological Activities of New Diphenylsulphone Derived Schiff Base Ligands and Their Co(II) Complexes

**DOI:** 10.3390/molecules27238576

**Published:** 2022-12-05

**Authors:** Kundalkesha D. Gaikwad, Panchsheela Ubale, Rahul Khobragade, Sachin Deodware, Pratibha Dhale, Mahadev R. Asabe, Rekha M. Ovhal, Pranav Singh, Prashant Vishwanath, Chandan Shivamallu, Raghu Ram Achar, Ekaterina Silina, Victor Stupin, Natalia Manturova, Ali A. Shati, Mohammad Y. Alfaifi, Serag Eldin I. Elbehairi, Shashikant H. Gaikwad, Shiva Prasad Kollur

**Affiliations:** 1 Department of Chemistry, Sangameshawar College, Solapur 413 001, India; 2 Chemistry Research Laboratory, Department of Chemistry, Shri Shivaji Mahavidyalaya, Solapur 413 411, India; 3 Department of Chemistry, N. K. Orchid College of Engineering and Technology, Solapur 413 002, India; 4 Department of Microbiology, Dr. Babasaheb Ambedkar Marathwada University, Sub Campus, Osmanabad 413 501, India; 5 Department of Chemistry, Walchand College of Art and Science, Solapur 413 006, India; 6 Department of Medicine, Kasturba Medical College, Manipal Academy of Higher Education, Udupi 576 104, India; 7 Centre for Excellence in Molecular Biology and Regenerative Medicine, Department of Biochemistry, JSS Medical College, JSS Academy of Higher Education and Research, Mysuru 570 015, India; 8 Department of Biotechnology and Bioinformatics, School of Life Sciences, JSS Academy of Higher Education and Research, Mysuru 570 015, India; 9 Division of Biochemistry, School of Life Sciences, JSS Academy of Higher Education and Research, Mysuru 570 015, India; 10 Department of Hospital Surgery, N.I. Pirogov Russian National Research Medical University, Moscow 117997, Russia; 11 Institute of Biodesign and Modeling of Complex Systems, I.M. Sechenov First Moscow State Medical University (Sechenov University), Moscow 119991, Russia; 12 Biology Department, Faculty of Science, King Khalid University, Abha 9004, Saudi Arabia; 13 Cell Culture Lab, Egyptian Organization for Biological Products and Vaccines (VACSERA Holding Company), 51 Wezaret El-Zeraa St., Giza 22311, Egypt; 14 School of Physical Sciences, Amrita Vishwa Vidyapeetham, Mysuru Campus, Mysuru 570 026, India

**Keywords:** 4,4′-diaminodiphenyl sulfone, Schiff base, Co(II) complex, antimicrobial, anticancer activity

## Abstract

The present work describes the chemical preparation of Schiff bases derived from 4,4′-diaminodiphenyl sulfone (L_1_–L_5_) and their Co(II) metal complexes. The evaluation of antimicrobial and anticancer activities against MCF-7 cell line and human lung cancer cell line A-549 was performed. The aforementioned synthesized compounds are characterized by spectroscopic techniques and elemental analysis confirms successful synthesis. The results from the above analytical techniques revealed that the complexes are in an octahedral geometry. The antimicrobial activity of the synthesized Schiff base ligands and their metal complexes under study was carried out by using the agar well diffusion method. The ligand and complex interactions for biological targets were predicted using molecular docking and high binding affinities. Further, the anticancer properties of the synthesized compounds are performed against the MCF-7 cell line and human lung cancer cell line A-549 using adriamycin as the standard drug.

## 1. Introduction

Coordination compounds play an important role in our daily lives, with applications ranging from biology to industry. Because of their high selectivity and target specificity in treating a variety of life-threatening diseases, coordination complexes are now replacing traditional organic drugs in biology. Metals, such as copper, calcium, iron, zinc, and cobalt, are important elements that have enormous biological activity when combined with certain metal proteins that help transport oxygen and are also useful in electronic transfer reactions and ion storage. Over the years, the chemistry of Schiff base complexes has progressed quickly, finding solutions in coordination and stereochemistry [1,2]. Cobalt 59 is the naturally occurring isotope. It is a highly essential trace element to all humans and animals [3,4,5].

Cobalt has several significant uses in a variety of fields. For instance, in vitamin B12 (cobalamin) form, it is important for a variety of biological functions. Cobalamin is essential for red blood cell formation, synthesis of DNA, and child growth and development. Co is also used as a catalyst in several reactions, in addition to both uses. Cobalt is also involved in the production of neurotransmitters, which are essential for the proper functioning of the nervous system, and thus the entire body. Furthermore, cobalt is needed for the formation of amino acids and few proteins in the myelin sheath in nerve cells. Glutamatase, dialdehydase, methionine synthease, mutase, and dipeptidase all contain cobalt. Further, Cobalt increases ATP turnover, which is essential for red blood cell development and animal growth [6,7].

Schiff base ligands and their coordination metal complexes are widely studied, owing to their easy and simple synthesis and good solubility in various solvents. Currently, such complexes are considered as successful models of biological compounds. One example is cobalt, which, despite its medicinal potential, is largely overlooked by pharmaceutical chemistry. Although there are some exceptional reviews of cobalt-based therapeutic research [8,9,10], the biological properties of cobalt complexes differ significantly based on the chelation strategy. Antimicrobials, anticancer agents, and protein aggregation inhibitors are only a few of the possible therapeutic activities of cobalt-Schiff base complexes. In addition, cobalt complexes are widely used as catalysts [11,12,13] in asymmetric hetero Diels Alder reactions [14] and in the asymmetric addition of organometallic reagents to aldehydes [15,16]. A lot of research has suggested that Co complexes have the potential to target cancer proteins. Schiff base Co(II) complexes, in particular, have better anticancer activity than cis-platin against cancer cells, e.g., HeLa and MCF-7 [17,18,19,20,21,22,23].

The present study deals with the preparation of Schiff bases and their Co(II) complexes using the known procedures. Further, the biological activities, viz. antimicrobial and anticancer activities, were performed to know the biological efficacy of the synthesized compounds.

## 2. Experimental

### 2.1. Materials and Methods

2-Hydroxy-1-naphathaldehyde, 5-bromo-2-hydroxybenzaldehyde, 4,4′-diaminodiphenylsulphone, 2-hydroxy-3-methoxy benzaldehyde, 5-chloro-2-hydroxybenzaldehyde, and 2-hydroxy-benzaldehyde were of AR grade from Sigma Aldrich. CoCl_2_.6H_2_O was purchased from Merck Chemical Ltd. (Mumbai, India). All chemicals/reagents were used as received. Euro EA CHNS elemental analyzer is used to determine the elemental composition of synthesized compounds. The functional groups in the molecules were determined by FT-IR spectroscopy in the range of 400–4000 cm^−1^ in KBr disc using Perkin-Elmer 1200 FT-IR spectrometer. The thermal response of the investigated complexes were taken using the Shimadzu DT-50 thermal analyzer with a heating rate of 10 °C/min in an atmosphere saturated with nitrogen. Electronic absorption of the compounds was investigated using a UV-Visible spectrophotometer (Shimadzu, UV-1800). ^1^H-NMR of the Schiff base ligands was recorded using Bruker Avance (400 MHz) ^1^H-NMR spectrometer. 

### 2.2. Chemical Synthesis

#### 2.2.1. Preparation of Schiff Base Ligands (L_1_–L_5_)

**Scheme 1:**
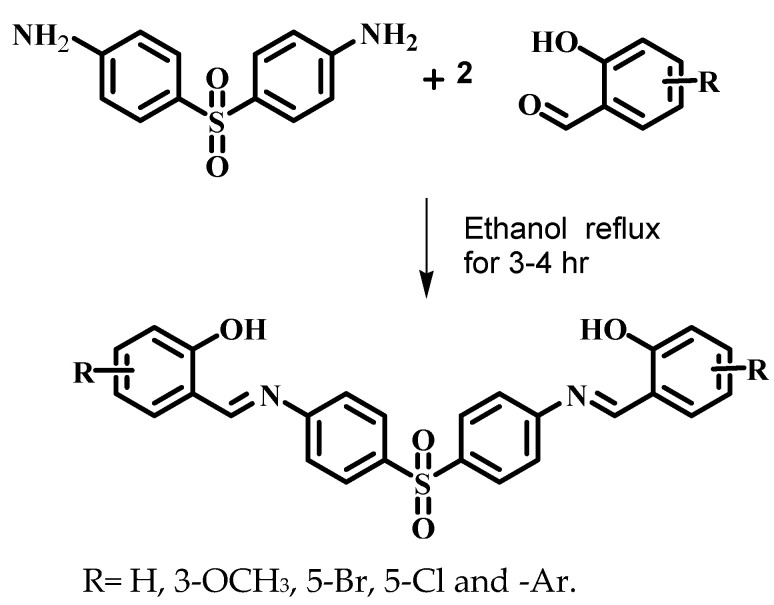
General synthetic route of Schiff base ligands (L_1_–L_5_).

The synthesis of series of Schiff bases is schematically represented in Scheme 1 and reported previously [23]. It is planned to synthesize tetradentate ligands consisting of ONNO, four donor sequence thus, 4,4′-diaminodiphenyl Sulfone (Dapsone) is made to react with 2,2-hydroxy-benzaldehyde, 2-hydroxy-1-naphathaldehyde, 2-hydroxy-3-methoxy benzaldehyde, 5-bromo-2-hydroxybenzaldehyde, and 5-chloro-2-hydroxybenzaldehyde in 1:1 ratio in ethanolic solution and the reaction mixture is refluxed for 4 h to yield Schiff bases (L_1_–L_5_). The physical and chemical characterization data of all the five Schiff base ligands are presented below:

##### 2,2′-(4,4′-Sulfonylbis(4,1-phenylene)bis(azan-1-yl-1-ylidene))bis(methan-1-yl-1-ylidene)-diphenol (L_1_)

Yield: 77%; mp: 231 °C; FT-IR (KBr, ν/cm^−1^): 3459 (OH), 3376 (Ar-C-H), 1615 (C=N), 1566 (C=C) 1274 (asymmetric -SO_2_-stretch), 1185 (symmetric -SO_2_- stretch); ^1^H-NMR (400 MHz, DMSO-*d*_6_) δ: 12.56 (1H, s, Ar-OH), 8.80 (1H, s, Azomethine), 6.55–8.05 (8H, m, Ar-H); Mass (*m/z*): 456 [M^+^]; Elemental analysis(%) for C_26_H_20_N_2_O_4_S: Expt.(calcd), C 68.43 (68.41), H 4.40 (4.39), N 6.21 (6.14).

##### 1,1′-(4,4′-Sulfonylbis(4,1-phenylene)bis(azan-1-yl-1-ylidene))bis(methan-1-yl-1-ylidene)-dinapthalen-2-ol (L_2_)

Yield: 82%; m.p.: 239 °C; FT-IR (KBr, ν/cm^−1^): 3434 (OH), 3222 (Ar-C-H), 1619 (C=N), 1544 (C=C), 1283 (asymmetric -SO_2_-stretch), 1186 (symmetric -SO_2_- stretch); ^1^H-NMR (400 MHz, DMSO-*d*_6_) δ: 12.83 (1H, s, Ar-OH), 8.72 (1H, s, Azomethine), 6.49–8.01 (10H, m, Ar-H); Mass (*m/z*): 556 [M^+^ + K]; Elemental analysis(%) for C_34_H_24_N_2_O_4_S: Expt.(calcd), C 73.42 (73.26), H 4.65 (4.48), N 5.13 (5.17).

##### 2,2′-(4,4′-Sulfonylbis(4,1-phenylene)bis(azan-1-yl-1-ylidene))bis(methan-1-yl-1-ylidene)bis-(4-bromophenol) (L_3_)

Yield: 68%; m.p.: 233 °C; FT-IR (KBr, ν/cm^−1^): 3427 (OH), 3230 (Ar-C-H), 1621 (C=N), 1547 (C=C), 1275 (asymmetric -SO_2_-stretch), 1182 (symmetric -SO_2_- stretch); ^1^H-NMR (400 MHz, DMSO-*d*_6_) δ: 12.89 (1H, s, Ar-OH), 8.68 (1H, s, Azomethine), 6.67–8.23 (7H, m, Ar-H); Mass (*m/z*): 614 and 616 [M^+^ and M^+^ + 2]; Elemental analysis(%) for C_26_H_18_Br_2_N_2_O_4_S: Expt.(calcd), C 50.18 (50.07), H 2.93 (2.86), N 4.56 (4.48).

##### 6,6′-(4,4′-Sulfonylbis(4,1-phenylene)bis(azan-1-yl-1-ylidene))bis(methan-1-yl-1-ylidene)bis-(2-methoxyphenol) (L_4_)

Yield: 80%; m.p.: 249 °C; FT-IR (KBr, ν/cm^−1^): 3432 (OH), 3236 (Ar-C-H), 1614 (C=N), 1579 (C=C), 1273 (asymmetric -SO_2_-stretch), 1187 (symmetric -SO_2_- stretch); ^1^H-NMR (400 MHz, DMSO-*d*_6_) δ: 12.89 (1H, s, Ar-OH), 8.70 (1H, s, Azomethine), 6.50–8.11 (7H, m, Ar-H), 3.81 (3H, s,-OCH_3_); Mass (*m/z*): 516 and 518 [M^+^ and M^+^ + 2]; Elemental analysis(%) for C_28_H_24_N_2_O_6_S: Expt.(calcd), C 65.16 (65.12), H 4.80 (4.74), N 5.53 (5.48).

##### 2,2′-(4,4′-Sulfonylbis(4,1-phenylene)bis(azan-1-yl-1-ylidene))bis(methan-1-yl-1-ylidene)bis-(4-chlorophenol) (L_5_)

Yield: 86%; mp: 260 °C; FT-IR (KBr, ν/cm^−1^): 3457 (OH), 3241 (Ar-C-H), 1627 (C=N), 1563 (C=C), 1268 (asymmetric -SO_2_-stretch), 1181 (symmetric -SO_2_- stretch); ^1^H-NMR (400 MHz, DMSO-*d*_6_) δ: 12.70 (1H, s, Ar-OH), 8.51 (1H, s, Azomethine), 6.67–7.97 (7H, m, Ar-H); Mass (*m/z*): 524 [M^+^]; Elemental analysis(%) for C_26_H_18_Cl_2_N_2_O_4_S: Expt.(calcd.), C 60.46 (60.33), H 3.48 (3.41), N 5.42 (5.35).

#### 2.2.2. Synthesis of Co(II) Complexes (C_1_–C_5_)

**Figure 1:**
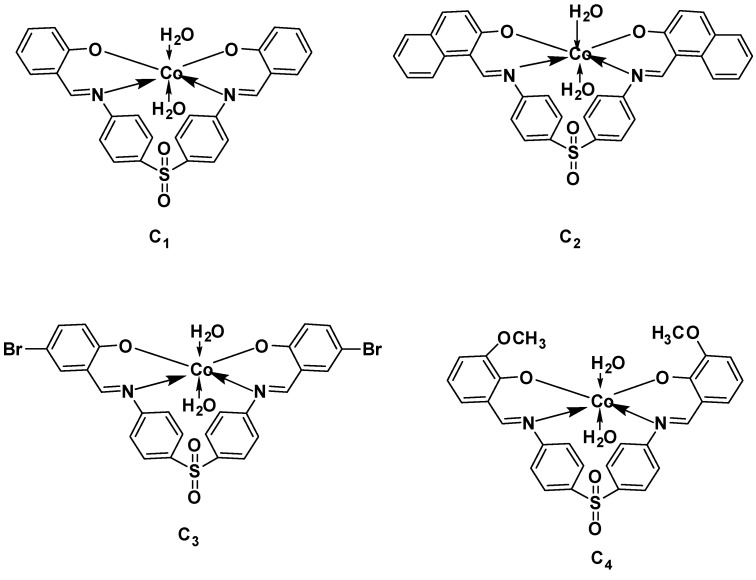

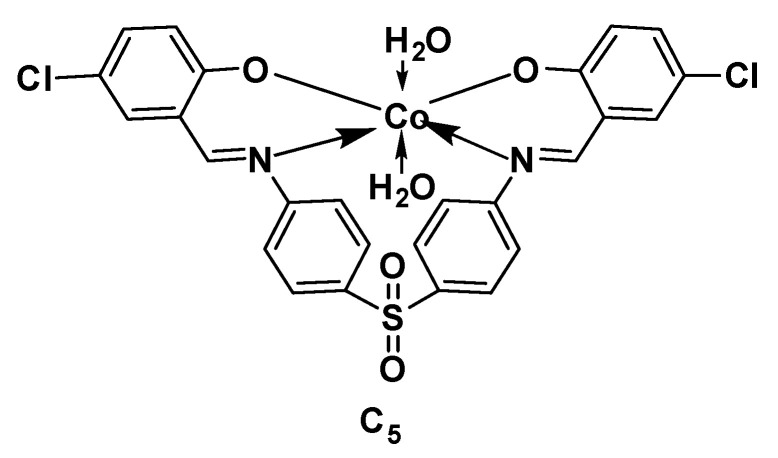
Proposed structure of synthesized Co(II) complexes (C_1_–C_5_).

An ethanolic solution of 0.1 M (4.56 g in 25 mL of ethanol) of the Schiff base ligand (L_1_) was added to a Co(II) chloride solution of 0.102 M (2.59 g in 10 mL of ethanol). The above reaction mixture was refluxed under continuous stirring for about 6 h. The progress of reaction was checked by TLC and spots were visualized under UV light. The precipitate obtained was then filtered, washed thoroughly with ethanol, and dried in desiccator over anhydrous CaCl_2_. Co(II) complexes with other Schiff bases (L_2_ (5.56 g), L_3_ (6.14 g), L_4_ (5.16 g) and L_5_ (5.24 g)) were synthesized using the above procedure. The synthesized complexes are characterized using physico-chemical techniques. The tentative structures of the prepared complexes are depicted in Figure 1.

### 2.3. Biological Assay

#### 2.3.1. Antimicrobial Studies

The antimicrobial activities of the synthesized Schiff base ligands and their cobalt metal complexes under study were carried out by using the agar well diffusion method. The Gram-positive pathogens *Bacillus subtilis* (ATCC 6633) and *Staphylococcus aureus* (ATCC6538) and Gram-negative pathogens *Klebsiella pneumonia (ATCC 13883)*, *Pseudomonas aeruginosa* (ATCC9027)*,* and *Escherichia coli* (ATCC 8739) were used in the biological potency evaluation. The antifungal activity of the compounds was tested against *Aspergillus niger (ATCC 16404)* and *Candida albicans* (ATCC 10231). Standard drugs used for the study were Ciprofloxacin and Nystatin for bacterial and fungal pathogens respectively [24].

The Muller Hinton agar plate surface was inoculated by the spread plate method with microbial inoculum over the entire agar surface [25]. Then, a hole with a diameter of 6–8 mm is punched aseptically with a sterile cork borer, and the volume of the desired antimicrobial compound dissolved in DMSO with desired concentration was introduced into the well. Then, agar plates were incubated under suitable conditions depending on bacterial/fungal species requirements. The antimicrobial agents diffuse in the agar medium and inhibit the growth of the microbial strain tested. The inhibition zones exhibit around the antimicrobial compound and are measured as the diameter of the zone of inhibition in millimeters (mm) [26,27].

#### 2.3.2. Molecular Docking Studies

Molecular docking is one of the most essential tools used in drug discovery due to its ability to predict, the conformation of small-molecule ligands within the appropriate target binding site with a substantial degree of accuracy. To find out the possible mode of action of the synthesized Schiff bases (L_1_–L_5_) and Co(II) complexes (C_1_–C_5_), molecular docking calculations of cysteine protease human cathepsin ki.-e.CDK7 (Cyclin dependent kinase-7) PDB ID: 1au2 were carried out using AutoDock 4.2. Lamarckian genetic algorithm. Auto Grid was used to define the active site and the grid size was set to 46 × 54 × 56 points which covers all the active site residues [20]. The grid spacing of 0.375 Å was centered on the selected flexible residues, which are the active sites of the CDK-7. The step size of 2.0 Å for translation and 10° for rotation were selected. The maximum number of energy evaluations was set to 2,500,000. A total of 10 runs were performed, and for each run, a maximum number of 27,000 genetic algorithms (GA) generations were performed on a single population of 150 individuals. The best-docked conformation among 10 conformations was obtained with the lowest binding energy values [28]. Interaction between cysteine protease human cathepsin ki.-e.CDK7 (Cyclin-dependent kinase-7) PDB ID: 1au2 with various ligands under study was visualized using molecular visualization tools such as Chimera (Pettersen et al., 2004). In addition, by using LigPlot+ tool hydrogen bonding and hydrophobic interactions were predicted for the complex [29].

#### 2.3.3. Anticancer Activities by SRB Assay

The cell lines were cultured and grown in an appropriate medium containing 10% fetal bovine serum (FBS) and 2 mM L-glutamine. In the presentation investigation of the screening experiment, 5000 cells/well were inoculated into 96 well microtiter plates in 100 µL. After cell inoculation, the microtiter plates were incubated at 37 °C, 5% CO_2_, 95% air, and 100% relative humidity for 24 h before the addition of experimental drugs. Experimental drugs were solubilized in a suitable solvent (100 mg/mL) and diluted to 1 mg/mL using double distilled water and frozen before use. During the time of drug addition, an aliquot of frozen concentrate (1 mg/mL) was thawed and diluted to 100, 200, 400, and 800 μg/mL with a complete medium containing the test sample. Aliquots of 10 µL of these different drug dilutions were added to the appropriate microtiter wells already containing 90 µL of the medium, resulting in the required final drug concentrations, i.e., 10, 20, 40, 80 μg/mL. After the addition of the test compound, the plates were incubated at standard conditions for 48 h, and the assay was terminated by the addition of cold TCA. Cells were fixed in situ by the gentle addition of 50 µL of cold 30% (*w/v*) TCA (final concentration, 10% TCA) and incubated for 60 min at 4 °C. The supernatant was discarded. The plates were washed five times with tap water and air dried. Sulforhodamine B (SRB) solution (50 µL) at 0.4% (*w/v*) in 1% acetic acid was added to each of the wells, and plates were incubated for 20 min at room temperature. After staining, the unbound dye was recovered, and the residual dye was removed by washing five times with 1% acetic acid. The plates were air dried. The bound stain was subsequently eluted with 10 mM trizma base, and the absorbance was read on a plate reader at a wavelength of 540 nm with 690 nm reference wavelength. Percent growth was calculated on a plate-by-plate basis for test wells relative to control wells. Percent growth was expressed as the ratio of the average absorbance of the test well to the average absorbance of the control wells × 100. This has been shown in the form of the equation below:

$$\mathrm{Percentage}\;\mathrm{growth}=\frac{\mathrm{Average}\;\mathrm{absorbance}\;\mathrm{of}\;\mathrm{the}\;\mathrm{cell}\;\mathrm{test}}{\mathrm{Average}\;\mathrm{absorbance}\;\mathrm{of}\;\mathrm{the}\;\mathrm{control}\;\mathrm{well}}\times 100$$

## 3. Results and Discussion

The elemental analysis data of synthesized Schiff bases and their complexes were found to be consistent with the expected result. The analytical and physical data of all the synthesized Schiff bases (L_1_ to L_5_) and their Co(II) complexes (C_1_ to C_5_) are given in Table 1. Theoretical and experimentally observed values of elemental analysis of compounds are in good agreement with the molecular formula. 

### 3.1. Electronic Spectral Studies amd Magnetic Moment Studies

**Figure 2:**
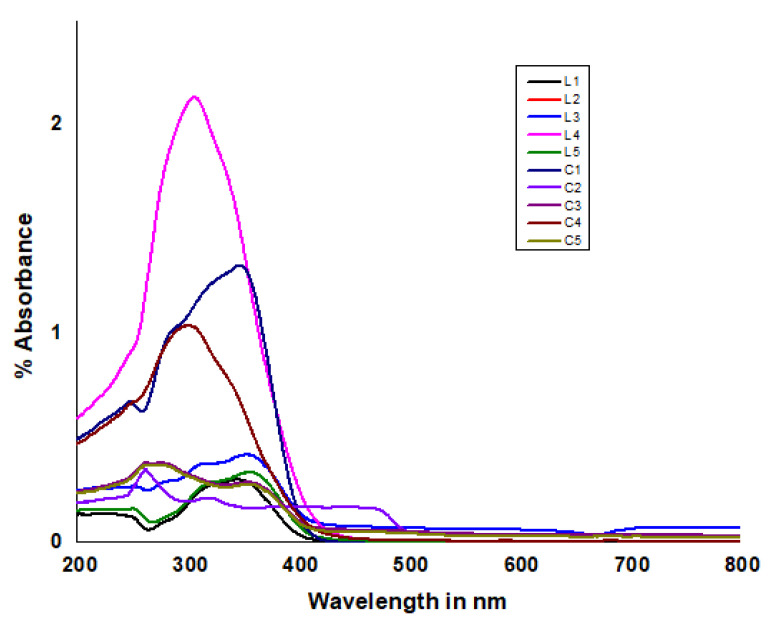
The UV-Vis bands (in nm) Schiff bases and complexes.

Electronic absorption spectra of Co(II) Schiff base complexes were recorded at room temperature in DMSO solution and the results are shown in Table 2 and Figure 2 respectively. The electronic spectra of C_1_, C_2_, and C_3_ showed three absorption bands. The first two were in the range 248–290 nm, which are due to π → π* transition of the aromatic ring and the third one was in the range 345–399 nm, which was assigned to n→ π* transition of azomethine group (C=N). The UV-Visible spectrum of C_4_ and C_5_ showed two absorption bands (Figure S24). First, one was at 255 and 245 nm, respectively, which was attributed to π → π* transition of the aromatic ring, and the second one at around 310 and 342 nm, which was assigned to n → π* transition of azomethine group (C=N) of Schiff base. The magnetic moment values of Co(II) complexes were observed in the range of 4.37–5.08 B.M. (Table 2) because of high-spin magnetic moments, corresponding to the unpaired electrons. It appears from the magnetic moment data of Co(II) complexes that they are paramagnetic in nature, and hence are of six-coordinate complexes [30].

### 3.2. FT- IR Spectral Studies

The formation of Co(II) complexes were confirmed as important shifts in azomethine group (C=N) and phenolic -OH bands by comparing the infrared spectroscopic data of metal complexes and their respective ligands. The IR spectral data of metal complexes are presented in Table 3. The stretching vibrational band observed around 1627–1614 cm^−1^ is a characteristic of the azomethine (C=N) nitrogen atom present in the free Schiff base ligand. Due to metal coordination, the expected typical imine band in the range 1620–1596 cm^−1^ was observed in the Co(II) complexes. In addition, the –OH stretching and bending vibrational frequencies of the substituted salicylaldehyde appeared in the region 3459–3427 cm^−1^. The disappearance of these two peaks in the spectra of all the Co(II) complexes indicates that the coordination takes place via the enolic –OH group. Furthermore, the presence of a broad band at around 3435–3367 cm^−1^ in the Co(II) complexes suggests the presence of coordinate water molecules to the central metal ion [31,32,33]. Additional evidence for the coordination of the azomethine nitrogen is the presence of ν(M-N) bands in the frequency range of 562–544cm^−1^ and ν(M-O) bands in the frequency range of 517–505 cm^−1^ [34,35,36]. It is noteworthy that the unchanged band position of sulphonyl groups suggests that sulphone is not taking part in the coordination (Figures S1–S9) [37,38].

### 3.3. ^1^H-NMR Spectral Studies

The ^1^H-NMR spectra of all the synthesized Schiff base ligands were recorded in DMSO solvent and expressed in ppm. The ^1^H-NMR spectra of the synthesized compounds exhibit signals due to aromatic protons as a multiplet at 6.49–8.23 ppm. In the ^1^H-NMR spectrum of the Schiff base ligands, a singlet observed downfield around 12.56–12.92 ppm, integrating for one proton, is assigned to –OH [39]. Similarly, the azomethine proton (attached to the carbon close to the nitrogen atom) appears around 8.68–8.80 ppm as a singlet signal [40]. Representative proton ^1^H-NMR spectra of L_1_, L_2_, L_3_, and L_5_ are shown in Figures S10–S13. The proton and carbon NMR of complexes are depicted in Figures S25–S30.

### 3.4. Thermogravimetric Analysis

**Figure 3:**
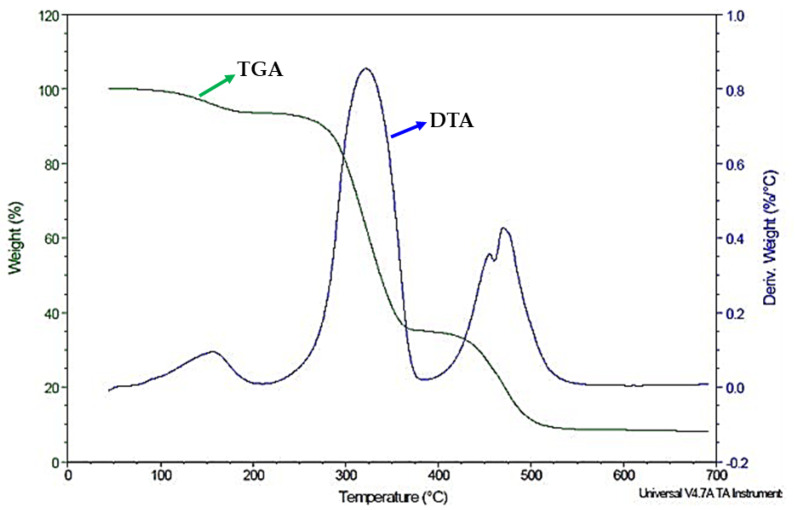
TGA-DTA graph of a C_1_ complex.

The loss of water molecules was observed below 150 °C, which is due to the presence of lattice water molecules [41,42]. This further tells us that the coordinated water molecules occupy some position in the coordination sphere of the central metal ion. These water molecules are more strongly bonded to the metal ions thus eliminated at the higher temperatures. A further rise in the temperature, leading to the loss of mass in a slow gradual manner, was observed, which may be caused due to decomposition of metal complexes by the fragmentation and thermal degradation of organic parts and at the end, the metal oxide was formed as residue [43]. The thermal data are provided in Table 4 and representative thermal graphs of Co(II) complex (C_1_) is shown in Figure 3.

### 3.5. Mass Spectral Studies

The mass spectra of all the Schiff base ligands exhibit parent ion peaks, due to their respective molecular ion (M^+^), corresponding to the molecular weight and confirming their molecular composition. The proposed molecular formula of these compounds was confirmed by comparing their molecular formula weights with the m/z values. The mass spectra of the Schiff base ligands are depicted in Figures S14–S18. In addition, the fragmentation pattern of L_1_ is depicted in Figure S23.

### 3.6. Antibacterial and Antifungal Activities

The antibacterial activity of synthesized Co(II) complexes was evaluated using different pathogens, including the Gram-positive *Bacillus Subtilis* and *Staphylococcus aureus*, Gram-negative *Klebsiella Species*, *E. coli*, and *Pseudomonas aeruginosac,* and fungal pathogens *Aspergillus niger* and *Candida albicans*. Antibacterial activity against DMSO and standard drugs Ciprofloxacin and Nystatin were also carried out. Bacterial and fungal pathogens showed the different zone of inhibitions against cobalt complexes (Figures S17–S20).

Considering the case of *Bacillus Subtilis*, for synthetic complex compounds (C_1_–C_5_) and DMSO, there was no zone of inhibition observed. The zone of inhibition for the standard drug Ciprofloxacin was 32 mm. However, in the case of *Staphylococcus aureus*, for synthetic compounds, C_1_, C_3_, C_4_, and C_5_, the zone of inhibition was 16, 16, 27, and 20, respectively. There was no zone of inhibition in the case of C_2_ and DMSO. The zone of inhibition for the standard drug Ciprofloxacin is 30 mm. Similarly, for *Klebsiella pneumonia*, for synthetic compounds, C_3_ and C_4_, the zone of inhibition was 8 and 16 mm, respectively. There was no zone of inhibition in the case of C_1_, C_2_, C_5_, and DMSO. The zone of inhibition for the standard drug Ciprofloxacin is 28 mm. Similarly, with *E. coli*, for synthetic compounds, C_2_, C_4_, and C_5_, the zone of inhibition was 15, 36, and 30 mm, respectively. There was no zone of inhibition in the case of C_1_, C_3_, and DMSO. The zone of inhibition for the standard drug Ciprofloxacin was 30 mm. In the case of *Pseudomonas aeroginosa,* for synthetic compounds, C_4_, and C_5_ the zone of inhibition was 16 and 12 mm, respectively. There was no zone of inhibition in the case of C_1_, C_2_, and DMSO. The zone of inhibition for the standard drug Ciprofloxacin was 28 mm (Figures S19–S22).

It is interesting to note that all the Schiff bases and their Co(II) complexes showed antibacterial activity against Gram-positive and Gram-negative bacteria (Table 5 and Table 6). This indicates the broad-spectrum ability of these compounds against the different pathogens. Ciprofloxacin as a standard drug and DMSO as a control was used for all bacterial species. 

**Figure 4:**
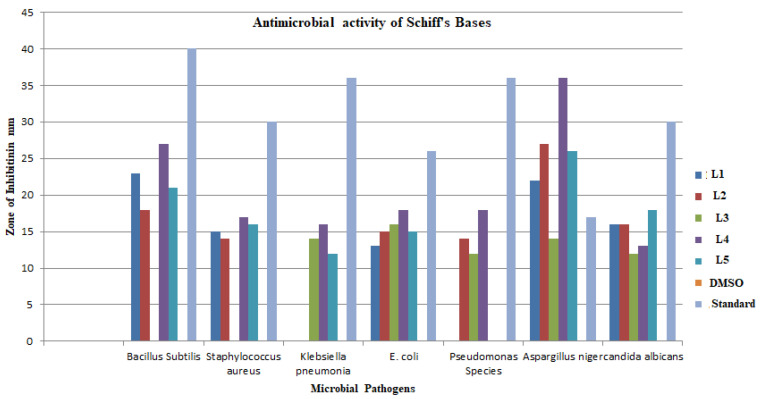
Graphical representation of antibacterial and antifungal activities of Schiff bases against bacterial and fungal pathogens.

The Schiff base L_4_ showed the highest antibacterial activity when compared with the other ligands (Figure 4). These compounds are not only active against bacteria, but also exhibit antifungal activity (Figures S19 and S20). Further, L_4_ exhibited strong antifungal activity against *Aspergillus niger (ATCC 16404)*. It is noteworthy that antifungal activities were higher than the standard antifungal drug Nystatin.

**Figure 5:**
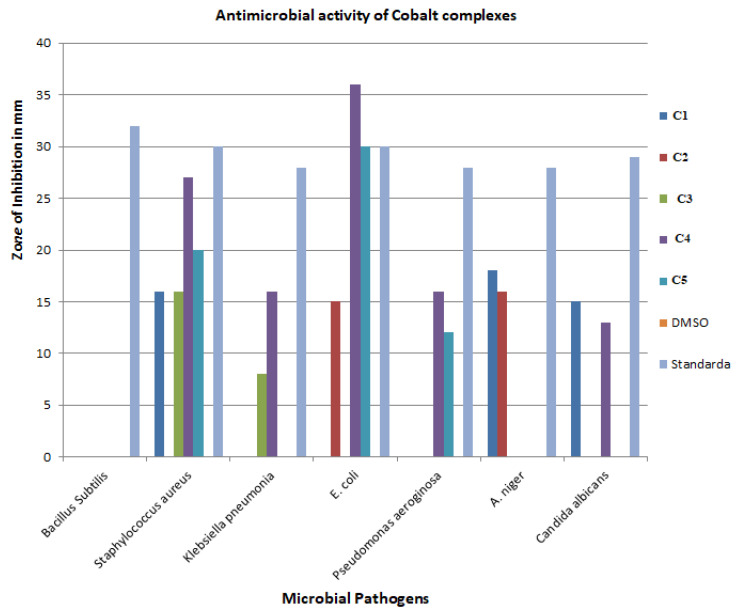
Graphical representation of antibacterial and antifungal activities of Co(II) complexes against bacterial and fungal pathogens.

On the other hand, it was observed from these results that the Co(II) complexes were less effective against both Gram-positive and Gram-negative. Moreover, only a few complexes show antifungal activity. There was no zone of inhibition shown by any cobalt complex against *Bacillus subtilis*. Out of five cobalt metal complexes, only C_4_ showed antibacterial activity against both Gram-positive, Gram-negative, and fungal pathogens (Figure 5). 

### 3.7. Molecular Docking Studies

To know the most preferred conformation of all Schiff base ligands with protein PDB ID:1au2, we performed the docking study of each ligand for 10 confirmations. The best-docked conformation among 10 conformations was obtained with the lowest binding energy. The binding values are −9.25 kcal/mol, −9.40 kcal/mol, −9.12 kcal/mol, −7.11 kcal/mol, and +285.63 kcal/mol for ligands CDK-7-L_1_, CDK-7-L_2_, CDK-7-L_3_, CDK-7-L_4_, and CDK-7-L_5_, respectively (Table 7). Further, the best-docked complexes were analyzed for hydrogen bonding interactions and hydrophobic interactions [44,45]. 

The results revealed that ARG179, ARG136, TYR190, and ARG188 residues of cysteine protease human cathepsin are involved in hydrogen bonding interactions. Whereas GLU99, ILE133, LEU134, ARG136, ASP137, LEU138, LYS139, PRO140, PHE156, GLY157, LEU158, PHE162, THR175, ARG176, TRP177, ARG179, LEU183, LEU184, ASP218, ARG188, MET189, and TYR190 residues involved in hydrophobic interactions. 

Compound L_2_ has the highest binding affinity followed by L_1_, L_3,_ and L_4_, whereas compound L_5_ has the lowest affinity among all the docked ligands, which is 285.63 K. Cal/Mol. Further, the L_2_ molecule showed interaction with amino acids, e.g., ARG188, TYR190, and PHE156, as well as hydrophobic and other interactions with LUE158, LEU134, ARG136, ASP137, THR175, ARG176, ARG179, and PHE162. The highest binding affinity of L_2_ than the other Schiff bases is mainly due to the involvement of ARG188 in the hydrogen bonding interaction and TYR190, LEU134, ARG136, ASP137, LEU158, THR175, ARG176, ARG179, and PHE162 in hydrophobic and other interactions with amino acid.

**Figure 6:**
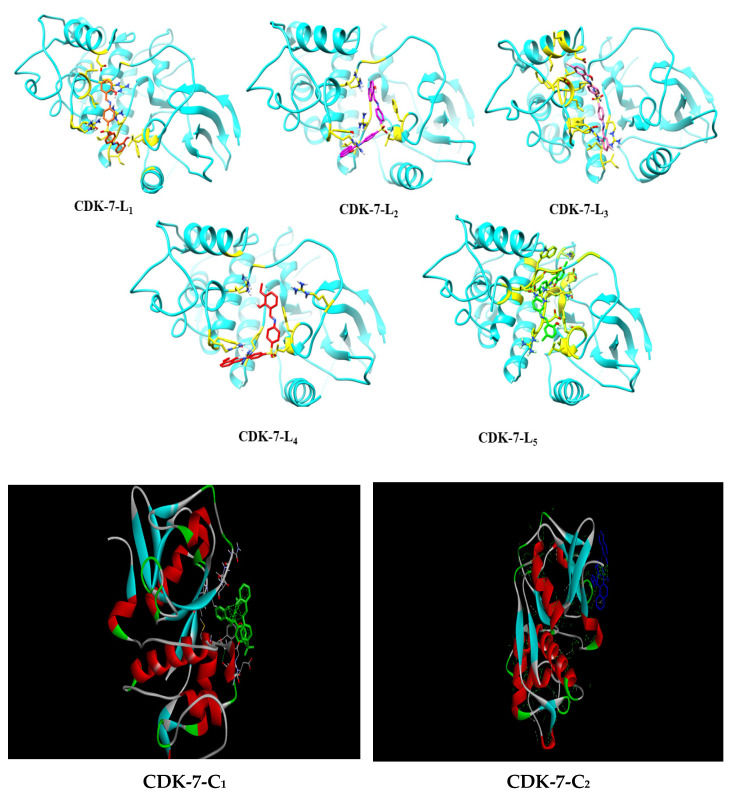
Binding interaction of Schiff base ligands (L_1_-L_5_) (top) and Co(II) complexes (C_1_–C_2_) with CDK-7 protein (bottom).

Using the data from docking interactions, 2D and 3D images are shown in Figure 6. Ligand L_1_ has the highest binding affinity followed by L_3_ L_4_, and L_5_ and whereas ligand L_5_ has the lowest affinity among all the docked Schiff base ligands, which is 285.63 kcal/mol. On the other hand, the docking results of Co(II) complexes revealed that GLN22, PHE23 SER161, ARG136, ARG179, TYR 190, and ARG188 residues of cysteine protease human cathepsin are involved in hydrogen bonding interactions. Whereas ALA198, LEU183, ALA180, VAL194, TYR178, LYS139, THR175, ASP137, LEU138, LYS41, ASN142, and PHE162, residues are involved in hydrophobic interactions, as depicted in Table 8.

The compound has C_2_ highest binding affinity, followed by C_3_, C_5_, and C_1_, whereas compound C_5_ has the lowest affinity among all the docked ligands, 2.47 K. Cal/Mol. Further, C_2_ complex and its interaction with amino acids, e.g., LEU134, PHE156, ILE133, GLU62, LUE158, ILE55, ARG136, ASP137, PHE162, ARG176, and ARG179, are involved in hydrophobic and other interactions. 

### 3.8. Effect of Ligands (L_1_–L_5_) and Their Complexes (C_1_–C_5_) on Antiproliferative Activity

**Figure 7:**
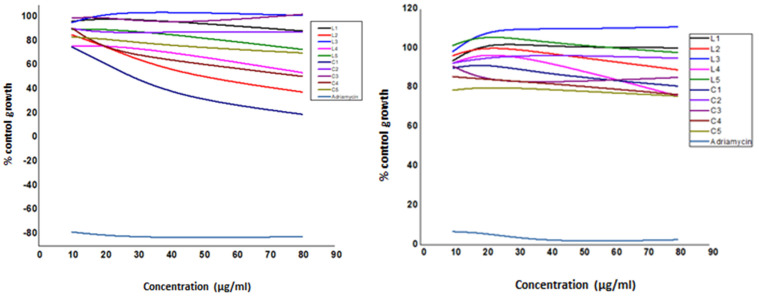
The growth curves of human breast cancer cell line MCF-7 (**left**) and human lung cancer cell line A-549 (**right**) of Schiff bases and their Co(II) complexes.

All the synthesized compounds were screened for their anticancer activity against human breast cancer cell line MCF-7 and human lung cancer cell line A-549. The growth curves of human breast cancer cell line MCF-7 and human lung cancer cell line A-549 of Schiff bases (L_1_–L_5_) and their Co(II) complexes (C_1_–C_5_) are represented in Figure 7. The anticancer activity was measured in vitro for the newly synthesized compounds against breast cancer cell line MCF-7and human lung cancer cell line A-549 using the Sulforhodamine B stain (SRB) assay method [43]. The average values for % control growth for the cell line MCF-7 and human lung cancer cell line A-549 are listed in Table 9 and Table 10.

The anticancer activity results based on average values for % control growth revealed that ligands and their Co(II) complexes are active at 20 and 40 μg/mL concentration. It is interesting to note that the complexes are more potent as compared to their respective ligand, except in the case of L_3_ and its complex. Moreover, it is noteworthy that the Schiff base ligands and Co(II) complexes are more active as compared to the standard drug adriamycin at all concentrations.

## 4. Conclusions

To sum up, the synthesized Schiff bases act as a tetradentate ligand and coordinated with the Co(II) ion through imine nitrogen and phenolic oxygen atoms. The binding of ligand to a metal ion was confirmed by elemental analysis, spectral studies (UV-Visible and FT-IR), and TGA measurements. The Co(II) complexes were found to exhibit octahedral geometry. All the Schiff bases (L_1_–L_5_) and their Co(II) complexes demonstrated moderate to good antimicrobial activity against the tested microbial species. The anticancer studies of the ligands and their Co(II)complexes showed significant activities against MCF-7 and human lung cancer cell line A-549 cancer cells. Particularly, the ligand L_3_ showed potential activity compared with the other tested compounds, which in turn was compared with the standard drug, adriamycin. Further, the molecular docking results revealed that ARG188, TYR190, and ARG136 residues of cysteine protease human cathepsin are involved in hydrogen bonding interactions.

## Figures and Tables

**Table 1:** Elemental analysis data of Schiff base ligands and their Co(II) complexes.

Compound	Mol. Formula	Mol. Wt.	M.P. (°C)	Elemental Analysis		
				C% Found (calc.)	H% Found (calc.)	N% Found (calc.)
L_1_	C_26_H_20_N_2_O_4_S	456	231	68.43(68.41)	4.40 (4.39)	6.21 (6.14)
L_2_	C_34_H_24_N_2_O_4_S	556	239	73.42 (73.26)	4.65 (4.48)	5.13(5.17)
L_3_	C_26_H_18_Br_2_N_2_O_4_S	614	233	50.18 (50.07)	2.93 (2.86)	4.56 (4.48)
L_4_	C_28_H_24_N_2_O_6_S	516	249	65.16(65.12)	4.80 (4.74)	5.53 (5.48)
L_5_	C_26_H_18_Cl_2_N_2_O_4_S	524	260	60.46 (60.33)	3.48 (3.41)	5.42 (5.35)
C_1_	C_26_H_22_N_2_O_6_SCo	549	347	57.19 (56.97)	4.08 (4.00)	5.37 (5.13)
C_2_	C_34_H_26_N_2_O_6_SCo	649	332	62.98 (62.89)	4.09 (4.03)	4.37 (4.31)
C_3_	C_26_H_20_N_2_O_6_SBr_2_Co	705	319	45.07 (44.56)	2.93 (2.86)	4.12 (3.99)
C_4_	C_28_H_26_N_2_O_8_SCo	609	352	56.23 (55.34)	4.37 (4.30)	4.77 (4.63)
C_5_	C_26_H_20_N_2_O_6_SCl_2_Co	617	324	51.76 (50.77)	3.32 (3.25)	4.66 (4.55)

**Table 2:** Electronic spectral and magnetic moment values for Co(II)-Schiff base metal complexes.

Complex	λ_max_ (nm)	Band Assignments	µeff. (BM)
	248	π-π*	
C_1_	289	π-π*	4.47
	345	n-π*	
	250	π-π*	
C_2_	283	π-π*	4.37
	399	n-π*	
	277	π-π*	
C_3_	290	π-π*	5.08
	375	n-π*	
C_4_	255 310	π-π* n-π*	4.86
	245	π-π*	
C_5_	342	n-π*	4.68

**Table 3:** FT-IR absorption bands (in cm^−1^) of the Schiffbases and their Co(II) complexes.

Compound	υ (OH/H_2_O)	υ (C=N)	υ (M-N)	*υ* (M-O)
L_1_	3459	1615	-	-
L_2_	3434	1619	-	-
L_3_	3427	1621	-	-
L_4_	3432	1614	-	-
L_5_	3457	1627	-	-
C_1_	3367	1614	544	514
C_2_	3433	1620	552	511
C_3_	3435	1610	562	510
C_4_	3402	1596	547	505
C_5_	3369	1601	552	517

**Table 4:** Stepwise thermal decomposition of Co(II) metal complexes.

Compound	Thermogravimetry (TG)		Mass Loss (%)		Decomposition Product Loss
	Stage	Temp (°C)	Found	Calculated	
C_1_	I	120–250	7.05	6.55	-2H_2_O
	II	250–440	80.74	82.91	Organic moiety
	III	440–1000	11.02	10.19	-CoO
C_2_	I	120–260	6.11	5.47	-2H_2_O
	II	260–410	81.03	82.98	Organic moiety
	III	410–1000	10.91	11.55	-CoO
C_3_	I	120–320	6.14	5.11	-2H_2_O
	II	320–425	83.69	84.26	Organic moiety
	III	425–1000	11.11	10.63	-CoO
C_4_	I	120–305	7.02	5.91	-2H_2_O
	II	305–410	82.31	81.79	Organic moiety
	III	410–1000	10.96	12.30	-CoO
C_5_	I	120–250	4.96	5.83	-2H_2_O
	II	250–450	83.03	82.03	Organic moiety
	III	450–1000	12.35	12.14	-CoO

**Table 5:** Antibacterial and Antifungal activities of Schiff bases ligands.

Microorganisms.	L_1_	L_2_	L_3_	L_4_	L_5_	DMSO	Standard ^a^
	Zone of growth inhibition in diameter (mm)						
Gram Positive							
*Bacillus subtilis* *(ATCC 6633)*	23	18	-	27	21	-	40
*Staphylococcus aureus* (ATCC 6538)	15	14	-	17	16	-	30
Gram negative							
*Klebsiella pneumonia (ATCC 13883)*	-	-	14	16	12	-	36
*Escherichia coli* (ATCC 8739)	13	15	16	18	15	-	26
*Pseudomonas aeruginosa* (ATCC 9027)	-	14	12	18	-	-	36
Fungal pathogens							
*Aspergillus niger (ATCC 16404)*	22	27	14	36	26	-	17
*Candida albicans* (ATCC 10231)	16	16	12	13	18	-	30

^a^ Standard used for antibacterial and antifungal activity was Ciprofloxacin and Nystatin respectively. “-”indicates a negative result.

**Table 6:** Antibacterial and Antifungal activities of Co(II) complexes.

Microorganisms	C_1_	C_2_	C_3_	C_4_	C_5_	DMSO	Standard ^a^
	Zone of growth inhibition in diameter (mm)						
Gram Positive							
*Bacillus Subtilis* *(ATCC 6633)*	-	-	-	-	-	-	32
*Staphylococcus Aureus* (*ATCC 6538*)	16	-	16	27	20	-	30
Gram Negative							
*Klebsiella pneumonia (ATCC 13883)*	-	-	08	16	-	-	28
*Escherichia coli* (*ATCC 8739*)	-	15	-	36	30	-	30
*Pseudomonas aeruginosa* (*ATCC 9027*)	-	-	-	16	12	-	28
Fungal Pathogens							
*Aspergillus niger (ATCC 16404)*	18	16	-	-	-	-	28
*Candida albicans* (*ATCC 10231*)	15	-	-	13	-	-	29

^a^ Standard used for antibacterial and antifungal activity was Ciprofloxacin and Nystatin. “-“ indicates a negative result.

**Table 7:** The Binding values of Schiff base ligands with CDK-7 protein.

Compound	Lowest Binding Affinity (kcal/mol)	RMSD from Reference Structure (Å)	Hydrogen Bond Interaction	Hydrogen Bond Length in Å	Hydrophobic and Other Interactions
CDK7-L_1_	−9.25	42.421	PHE136	2.85	LEU158, LEU134, ARG179, LEU183, ILE133, ARG136, LEU184, ASP218
			ARG188	2.81 2.84	
			TYR190	2.61	
CDK7-L_2_	−9.40	35.068	ARG 188	2.59 2.73 3.08 3.13	TYR190, LEU134, ARG136, ASP137, LEU 158, THR175, ARG176, ARG179, PHE162
CDK7-L_3_	−9.12	37.191	ARG179	2.79	LEU134, ARG136, LEU 183, LEU184, ASP218
			TYR190	3.02	
CDK7-L_4_	−7.11	43.983	ARG136	2.89 2.95 3.27	ARG179, PHE162, LEU158, ARG188, MET189, LEU134
			TYR190	3.27 2.6	
CDK7-L_5_	+285.63	35.985	ARG179	2.64	GLU99, LYS139, PRO140, TRP177, THER175, ARG176, ARG136, LEU183, LEU138, LEU158, GLY157, PHE162
			ASN141	3.05	

**Table 8:** The Binding values of Co(II) metal complexes with CDK-7 protein.

Compounds	Lowest Binding Affinity (kcal/mol)	RMSD from Reference Structure(Å)	Hydrogen Bond Interaction	Hydrogen Bond Length in Å	Hydrophobic and Other Interactions
CDK-7-C_1_	−2.47	39.661	GLN22	2.46	ALA198, LEU183, ALA180, VAL194, TYR178, LYS139, THR175, ASP137, LEU138, LYS41, ASN142, PHE162
			PHE23	3.22	
			SER161	2.56 2.94	
			ARG136	2.36	
			ARG179	2.64	
CDK-7-C_2_	−8.09	42.083	TYR190	2.80	LEU134, PHE156, ILE133, GLU62, LEU158, ILE55 ARG136, ASP137, PHE162, ARG176, ARG179
CDK-7-C_3_	−4.18	43.033	ARG188	3.00 2.53	MET189, ILE55, PHE162, LEU134
			TYR190	3.25	
CDK-7-C_4_	−3.00	41.939	ARG188	3.08 2.98	MET189, PHE162, ILE55, GLY163, PRO165
			TYR190	2.79 2.81	
CDK-7-C_5_	−3.92	42.169	ARG188	2.99	MET189, LEU134, LEU158, PHE162, ILE55
			TYR190	2.90 2.56	

**Table 9:** Average values for % control growth for the cell line MCF-7.

Concentration (μg/mL)	Average Values for % Control Growth										
	L_1_	L_2_	L_3_	L_4_	L_5_	C_1_	C_2_	C_3_	C_4_	C_5_	Adriamycin
10	96.3	85.0	95.2	75.7	89.7	74.9	90.3	99.1	90.6	83.2	−78.5
20	98.9	75.7	103.1	76.3	89.9	61.4	86.4	99.9	72.5	81.7	−81.6
40	96.5	53.3	104.2	71.0	85.6	33.7	87.4	93.5	63.8	75.8	−83.6
80	88.2	37.5	101.0	53.6	73.0	19.1	87.6	102.0	50.5	70.0	−82.5

**Table 10:** Average values for % control growth for the lung cell line A-549.

Concentration (μg/mL)	Average Values for % Control Growth										
	L_1_	L_2_	L_3_	L_4_	L_5_	C_1_	C_2_	C_3_	C_4_	C_5_	Adriamycin
10	93.6	96.2	98.3	92.3	101.4	89.9	92.4	90.8	85.5	78.6	6.6
20	103.4	101.5	110.1	98.2	107.3	92.4	95.3	83.2	84.2	80.3	5.9
40	100.5	97.3	109.6	93.1	102.5	86.5	96.8	82.4	82.1	79.0	1.1
80	100.2	89.0	110.8	75.7	97.8	80.7	94.9	85.1	76.4	75.6	2.5

## Data Availability

Not available.

## Supplementary Materials

The following supporting information can be downloaded at: https://www.mdpi.com/article/10.3390/molecules27238576/s1, Figures S1–S9: FT-IR spectra of ligands and complexes; Figures S10–13: ^1^H-NMR of Schiff base ligands; Figures S14–S18: Mass spectra of ligands; Figures S19–S22: Antimicrobial activities of ligands and complexes; Figure S23: Mass fragmentation pattern of L_1_; Figure S24: UV-Vis absorption spectra of complexes; Figures S25–S30: ^1^H and ^13^C-NMR od complexes.

## References

[1] Khandar A.A., Nejati K., Rezvani Z. (2005). Syntheses, Characterization and Study of the Use of Cobalt (II) Schiff–Base Complexes as Catalysts for the Oxidation of Styrene by Molecular Oxygen. Molecules.

[2] Martell A.E., Sawyer D.T. (1998). Oxygen-Co Complexes and Oxygen Activation by Transition Metals.

[3] Nagata T., Yorozu K., Yamada T., Mukaiyama T. (1995). Enantioselective Reduction of Ketones with Sodium Borohydride, Catalyzed by Optically Active (β-Oxoaldiminato)cobalt(II) Complexes. Angew. Chem. Int. Ed..

[4] Estiú G.L., Jubert A.H., Costamagna J., Vargas J. (1996). Quantum Chemical Calculations of the Structures and Electronic Properties of N,N‘- Bis(3,5-dibromosalicylidene)-1,2-diaminobenzene and Its Cobalt(II) Complex. Origin of the Redox Activity of the Cobalt Complex. Inorg. Chem..

[5] Zhang Y.-L., Ruan W.-J., Zhao X.-J., Wang H.-G., Zhu Z.-A. (2003). Synthesis and characterization of axial coordination cobalt(III) complexes containing chiral Salen ligands. Polyhedron.

[6] Cini R., Moore S.J., Marzilli L.G. (1998). Strong Trans Influence Methoxymethyl Ligand in B12 Cobaloxime and Imine/Oxime Model Complexes:  Structural, Spectroscopic, and Molecular Mechanics Investigations. Inorg. Chem..

[7] Sethi R., Ahuja M. (2016). Synthesis, Characterization and Antibacterial Activity of Cobalt Complex of 2-Pyrazoline with Pyridinyl MoietyInt. J. Pharm. Technol. Res..

[8] Ghosh P., Chowdhury A.R., Saha S.K., Ghosh M., Pal M., Murmu N.C., Banerjee P. (2015). Synthesis and characterization of redox non-innocent cobalt(III) complexes of a O,N,O donor ligand: Radical generation, semi-conductivity, antibacterial and anticancer activities. Inorganica Chim. Acta.

[9] Ghosh M., Layek M., Fleck M., Saha R., Bandyopadhyay D. (2015). Synthesis, crystal structure and antibacterial activities of mixed ligand copper(II) and cobalt(II) complexes of a NNS Schiff base. Polyhedron.

[10] Singh U., Malla A.M., Bhat I.A., Ahmad A., Bukhari M.N., Bhat S., Anayutullah S., Hashmi A.A. (2016). Synthesis, molecular docking and evaluation of antifungal activity of Ni(II),Co(II) and Cu(II) complexes of porphyrin core macromolecular ligand. Microb. Pathog..

[11] Ubale P., Mokale S., More S., Waghamare S., More V., Munirathinam N., Dilipkumar S., Das R.K., Reja S., Helavi V.B. (2021). Evaluation of in vitro anticancer, antimicrobial and antioxidant activities of new Cu(II) complexes derived from 4(3H)-quinazolinone: Synthesis, crystal structure and molecular docking studies. J. Mol. Struct..

[12] Ashok U.P., Kollur S.P., Anil N., Arun B.P., Jadhav S.N., Sarsamkar S., Helavi V.B., Srinivasan A., Kaulage S., Veerapur R. (2020). Preparation, Spectroscopic Characterization, Theoretical Investigations, and In Vitro Anticancer Activity of Cd(II), Ni(II), Zn(II), and Cu(II) Complexes of 4(3H)-Quinazolinone-Derived Schiff Base. Molecules.

[13] Nesterov D.S., Nesterova O.V. (2018). Polynuclear Cobalt Complexes as Catalysts for Light-Driven Water Oxidation: A Review of Recent Advances. Catalysts.

[14] Mil’Grom A.E., Chegolya A.S., Filippova A.V., Vladyko G.V., Karako N.I. (1984). Synthesis and antivirus activity of unprotonated complex salts of amino acids with nickel and cobalt. Pharm. Chem. J..

[15] Zhang H., Huang J., Meng F. (2021). Cobalt-catalyzed diastereo- and enantioselective allyl addition to aldehydes and α-ketoesters through allylic C–H functionalization. Cell Rep. Phys. Sci..

[16] Wang N., Lin Q.Y., Feng J., Zhao Y.L., Wang Y.J., Li S.K. (2010). Crystal structures, DNA interaction and antiproliferative activities of the cobalt(II) and zinc(II) complexes of 2-amino- 1,3,4-thiadiazole with demethylcantharate. Inorg. Chim. Acta.

[17] Munteanu C.R., Suntharalingam K. (2015). Advances in cobalt complexes as anticancer agents. Dalton Trans..

[18] Cavicchioli M., Zaballa A.M.L., de Paula Q.A., Prieto M.B., Oliveira C.C., Civitareale P., Ciriolo M.R., Ferreira A.M.D.C. (2019). Oxidative Assets Toward Biomolecules and Cytotoxicity of New Oxindolimine-Copper(II) and Zinc(II) Complexes. Inorganics.

[19] King A.P., Gellineau H.A., Ahn J.-E., MacMillan S.N., Wilson J.J. (2017). Bis(thiosemicarbazone) Complexes of Cobalt(III). Synthesis, Characterization, and Anticancer Potential. Inorg. Chem..

[20] Banerjee S., Chakravarty A.R. (2015). Metal Complexes of Curcumin for Cellular Imaging, Targeting, and Photoinduced Anticancer Activity. Accounts Chem. Res..

[21] Wasylenko D.J., Ganesamoorthy C., Borau-Garcia J., Berlinguette C.P. (2011). Electrochemical evidence for catalytic water oxidation mediated by a high-valent cobalt complex. Chem. Commun..

[22] Cahiez G., Moyeux A. (2010). Cobalt-Catalyzed Cross-Coupling Reactions. Chem. Rev..

[23] Gaikwad K.D., Khobragade R.M., Deodware S.A., Ubale P.A., Dhale P.C., Ovhal R.M., Shivamallu C., Ankegowda V.M., Raghavendra H., Gaikwad S.H. (2022). Chemical synthesis, spectral characterization and biological activities of new diphenylsulphone derived Schiff base ligand and their Ni(II) complexes. Results Chem..

[24] Waghmare S.R., Mulla M.N., Marathe S.R., Sonawane K.D. (2015). Ecofriendly production of silver nanoparticles using Candida utilis and its mechanistic action against pathogenic microorganisms. 3 Biotech.

[25] El-Sonbati A., Mahmoud W., Mohamed G.G., Diab M., Morgan S., Abbas S. (2019). Synthesis, characterization of Schiff base metal complexes and their biological investigation. Appl. Organomet. Chem..

[26] Devi J., Yadav M., Kumar A., Kumar A. (2018). Synthesis, characterization, biological activity, and QSAR studies of transition metal complexes derived from piperonylamine Schiff bases. Chem. Pap..

[27] Koçoğlu S., Ogutcu H., Hayvalı Z. (2019). Photophysical and antimicrobial properties of new double-armed benzo-15-crown-5 ligands and complexes. Res. Chem. Intermed..

[28] Mirdya S., Drew M.G., Chandra A.K., Banerjee A., Frontera A., Chattopadhyay S. (2020). A series of hydrogen bond mediated dinuclear nickel(II) complexes with reduced Schiff base ligands: An insight into the nature of their short intermolecular hydrogen bonds. Polyhedron.

[29] Bikas R., Mirzakhani P., Noshiranzadeh N., Sanchiz J., Krawczyk M.S., Kalofolias D.A., Lis T. (2020). Synthesis, crystal structure and magnetic properties of a pentanuclear Mn (III) cluster with 1, 2, 4-triazole based Schiff base ligand. Inorg. Chim. Acta.

[30] Nejo A.A., Kolawole G.A., Nejo A.O. (2010). Synthesis, characterization, antibacterial, and thermal studies of unsymmetrical Schiff-base complexes of cobalt(II). J. Coord. Chem..

[31] Saghatforoush L.A., Chalabian F., Aminkhani A., Karimnezhad G., Ershad S. (2009). Synthesis, spectroscopic characterization and antibacterial activity of new cobalt(II) complexes of unsymmetrical tetradentate (OSN2) Schiff base ligands. Eur. J. Med. Chem..

[32] Sebastian M., Arun V., Robinson P.P., Leeju P., Varghese D., Varsha G., Yusuff K.K.M. (2009). Synthesis, characterization, and structure of a new cobalt(II) Schiff-base complex with quinoxaline- 2-carboxalidine-2-amino-5-methylphenol. J. Coord. Chem..

[33] Barakat A., Soliman S.M., Ali M., Elmarghany A., Al-Majid A.M., Yousuf S., Ul-Haq Z., Choudhary M.I., El-Faham A. (2019). Synthesis, crystal structure, evaluation of urease inhibition potential and the docking studies of cobalt(III) complex based on barbituric acid Schiff base ligand. Inorg. Chim. Acta.

[34] Ghosh K., Dutta T., Drew M.G., Frontera A., Chattopadhyay S. (2020). A combined experimental and theoretical study on an ionic cobalt(III/II) complex with a Schiff base ligand. Polyhedron.

[35] Luo X.-Q., Liu Q.-R., Han Y.-J., Xue L.-W. (2020). Syntheses, X-ray Single Crystal Structures and Biological Activities of Cobalt(III) Complexes with Reduced Schiff Base Ligands. Acta Chim. Slov..

[36] Hricovíniová Z., Hricovíni M., Kozics K. (2017). New series of quinazolinone derived Schiff’s bases: Synthesis, spectroscopic properties and evaluation of their antioxidant and cytotoxic activity. Chem. Pap..

[37] Tang B., Yue T., Wu J., Dong Y., Ding Y., Wang H. (2004). Rapid and sensitive spectrofluorimetric determination of trace amount of Cr(III) with o-vanillin-8-aminoquinoline. Talanta.

[38] Tas E., Kilic A., Durgun M., Yilmaz I., Ozdemir I., Gurbuz N. (2009). Mono- and dinuclear Pd(II) complexes of different salicylaldimine ligands as catalysts of transfer hydrogenation of nitrobenzene with cyclohexene and Suzuki–Miyaura coupling reactions. J. Organomet. Chem..

[39] Venkatachalam G., Ramesh R. (2006). Ruthenium(III) bis-bidentate Schiff base complexes mediated transfer hydrogenation of imines. Inorg. Chem. Commun..

[40] Kalkhambkar R.G., Kulkarni G.M., Kamanavalli C.M., Premkumar N., Asdaq S., Sun C.M. (2008). Synthesis and biological activities of some new fluorinated coumarins and 1-aza coumarins. Eur. J. Med. Chem..

[41] Ashok U.P., Kollur S.P., Arun B.P., Sanjay C., Suresh K.S., Anil N., Baburao H.V., Markad D., Castro J.O., Frau J. (2020). In vitro anticancer activity of 4(3H)- Quinazolinone derived Schiff base and its Cu(II), Zn(II) and Cd(II) complexes: Preparation, X-ray structural, spectral characterization and theoretical investigations. Inorg. Chim. Acta.

[42] Vinusha H., Kollur S.P., Revanasiddappa H., Ramu R., Shirahatti P.S., Prasad M.N., Chandrashekar S., Begum M. (2019). Preparation, spectral characterization and biological applications of Schiff base ligand and its transition metal complexes. Results Chem..

[43] Razali N., Razab R., Junit S.M., Aziz A.A. (2008). Radical scavenging and reducing properties of extracts of cashew shoots (Anacardium occidentale). Food Chem..

[44] Dundas J., Ouyang Z., Tseng J., Binkowski A., Turpaz Y., Liang J. (2006). CASTp: Computed atlas of surface topography of proteins with structural and topographical mapping of functionally annotated residues. Nucleic Acids Res..

[45] Laskowski R.A., Swindells M.B. (2011). LigPlot+: Multiple ligand–protein interaction diagrams for drug discovery. J. Chem. Inf. Model..

