# Supplementary material for: Preparation, Characterization and In Vitro Biological Activities of New Diphenylsulphone Derived Schiff Base Ligands and Their Co(II) Complexes
Source: Molecules. 2022 Dec 5;27(23):8576. doi: 10.3390/molecules27238576 (PMC9741402; doi:10.3390/molecules27238576)
Supplement: Supplementary file 1 [file molecules-27-08576-s001.zip › molecules-2023508-supplementary.pdf]

Supporting Information

Preparation, Characterization and *in vitro* Biological Activities of New Diphenylsulphone Derived Schiff Base Ligands and Their Co(II) Complexes

Kundalkesha D. Gaikwad [1,2], Panchsheela Ubale [3], Rahul Khobragade [4], Sachin Deodware [2], Pratibha Dhale [2], Mahadev R. Asabe [5], Rekha M. Ovhal [5], Pranav Singh [6], Prashant Vishwanath [7], Chandan Shivamallu [8], Raghu Ram Achar [9], Ekaterina Silina [10,11], Victor Stupin [10], Natalia Manturova [10], Ali A. Shati [12], Mohammad Y. Alfaifi [12], Serag Eldin I. Elbehairi [12,13], Shashikant H. Gaikwad [2,*] and Shiva Prasad Kollur [14,*]

1 Department of Chemistry, Sangameshawar College, Solapur 413 001, India
2 Chemistry Research Laboratory, Department of Chemistry, Shri Shivaji Mahavidyalaya, Barshi, Solapur 413 411, India
3 N.K. Orchid College of Engineering and Technology, Solapur 413 002, India
4 Department of Microbiology, Dr. Babasaheb Ambedkar Marathwada University, Sub Campus, Osmanabad 413 501, India
5 Department of Chemistry, Walchand College of Art and Science, Solapur 413 006, India
6 Department of Medicine, Kasturba Medical College, Manipal Academy of Higher Education, Manipal, Udupi 576 104, India
7 Centre for Excellence in Molecular Biology and Regenerative Medicine, Department of Biochemistry, JSS Medical College, JSS Academy of Higher Education and Research, Mysuru 570 015, India
8 Department of Biotechnology and Bioinformatics, School of Life Sciences, JSS Academy of Higher Education and Research, Mysuru 570 015, India
9 Division of Biochemistry, School of Life Sciences, JSS Academy of Higher Education and Research, Mysuru 570 015, India
10 Department of Hospital Surgery, N.I. Pirogov Russian National Research Medical University, Moscow 117997, Russia
11 Institute of Biodesign and Modeling of Complex Systems, I.M. Sechenov First Moscow State Medical University (Sechenov University), Moscow 119991, Russia
12 Biology Department, Faculty of Science, King Khalid University, Abha 9004, Saudi Arabia
13 Cell Culture Lab, Egyptian Organization for Biological Products and Vaccines (VACSERA Holding Company), 51 Wezaret El-Zeraa St., Giza 22311, Egypt
14 School of Physical Sciences, Amrita Vishwa Vidyapeetham, Mysuru Campus, Mysuru 570 026, India
* Correspondence: rasayanshg@gmail.com (S.H.G.); shivachemist@gmail.com (S.P.K.)

Contents:

Figure S1: FT-IR spectrum of L_1.

Figure S2: FT-IR spectrum of L_2.

Figure S3: FT-IR spectrum of L_4.

Figure S4: FT-IR spectrum of L_5.

Figure S5: FT-IR spectrum of C_1.

Figure S6: FT-IR spectrum of C_2.

Figure S7: FT-I spectrum of C_3.

Figure S8: FT-IR spectrum of C_4.

Figure S9: FT-IR spectrum of C_5.

Figure S10: ^{1}H-NMR spectrum of L_1.

Figure S11: ^{1}H-NMR spectrum of L_2.

Figure S12: ^{1}H-NMR spectrum of L_4.

Figure S13: ^{1}H-NMR spectrum of L_5.

Figure S14: Mass spectrum of L_1.

Figure S15: Mass spectrum of L_2.

Figure S16: Mass spectrum of L_3.

Figure S17: Mass spectrum of L_4.

Figure S18: Mass spectrum of L_5.

Figure S19: Ni-and ligands Antibacterial activity against *Staphylococcus aureus.*

Figure S20: Ni-complex Antifungal activity against *Aspergillus niger.*

Figure S21: Ligand-Antifungal activity against *Aspergillus niger.*

Figure S22: Ni and Ligands Antibacterial activity against *Bacillus subtilis.*

Figure S23: Mass fragmentation pattern of L_1.

Figure S24: UV-Visible absorption spectra of complexes.

Figure S25: ^{1}H-NMR spectrum of C_1.

Figure S26: ^{1}H-NMR spectrum of C_2.

Figure S27: ^{1}H-NMR spectrum of C_3.

Figure S28: ^{13}C-NMR spectrum of C_1.

Figure S29: ^{13}C-NMR spectrum of C_2.

Figure S30: ^{13}C-NMR spectrum of C_3.

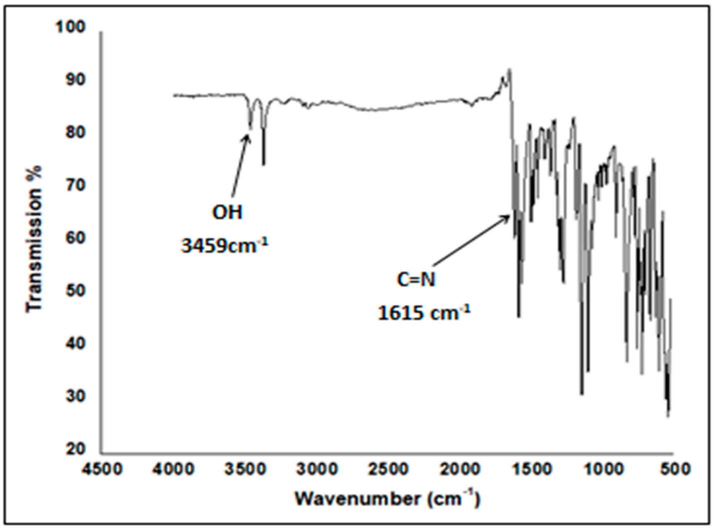

Figure S1: FT-IR spectrum of L_1.

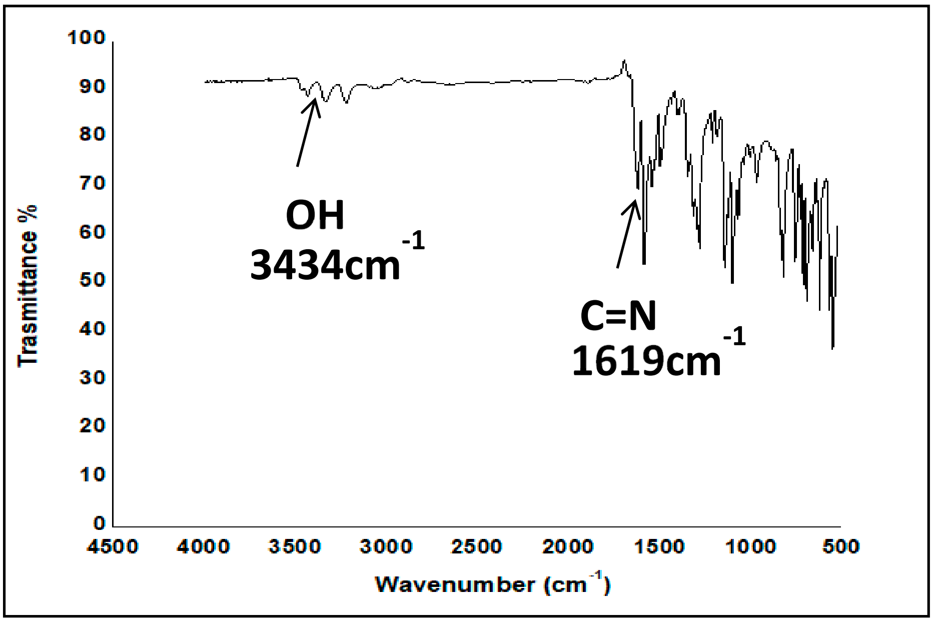

Figure S2: FT-IR spectrum of L_2.

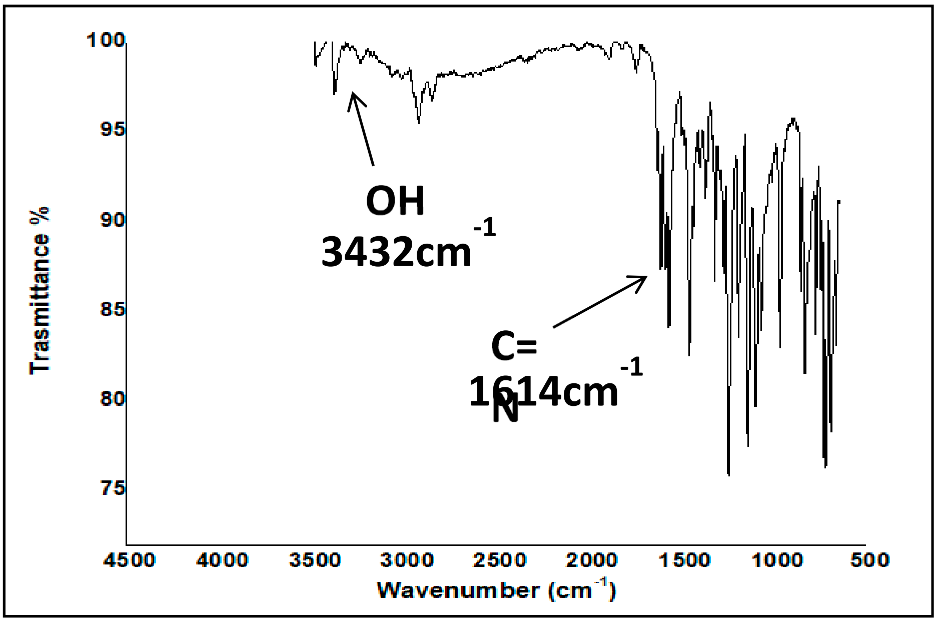

Figure S3: FT-IR spectrum of L_4.

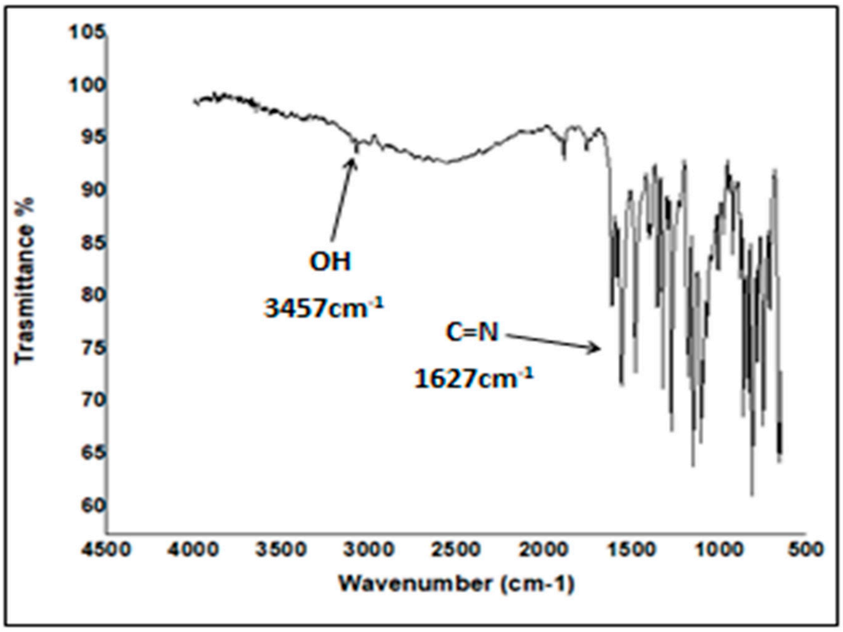

Figure S4: FT-IR spectrum of L_5.

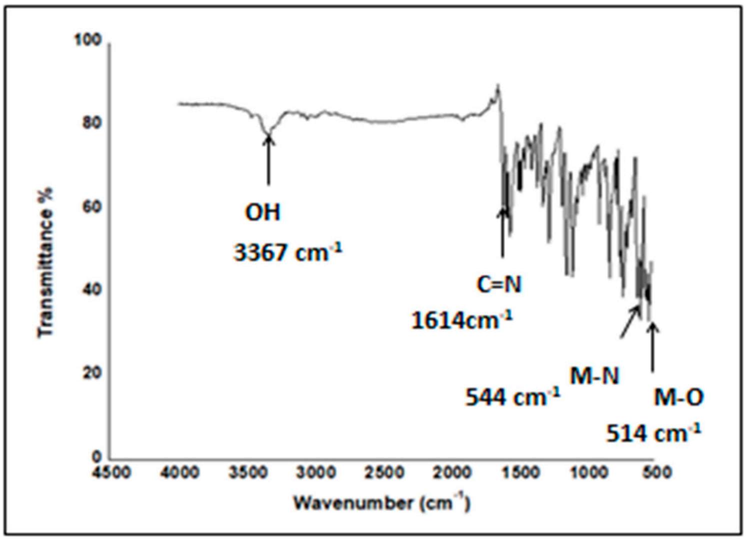

Figure S5: FT-IR spectrum of C_1.

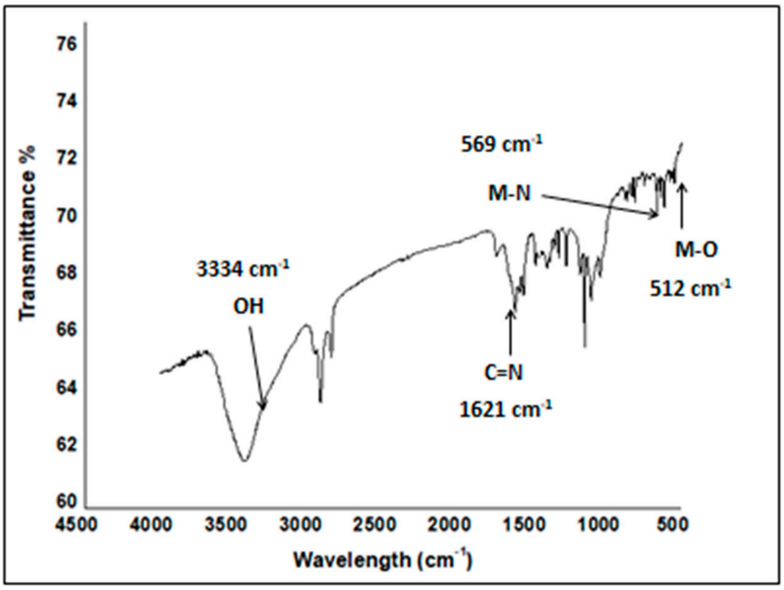

Figure S6: FT-IR spectrum of C_2.

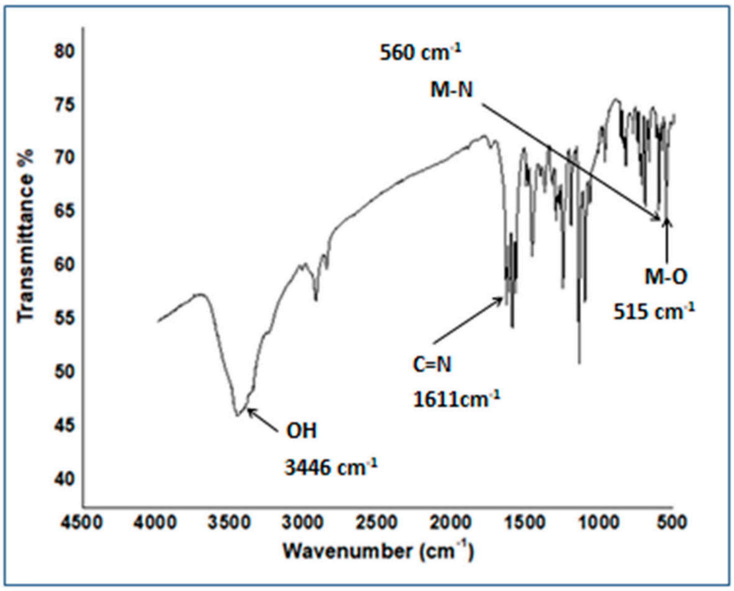

Figure S7: FT-IR spectrum of C_3.

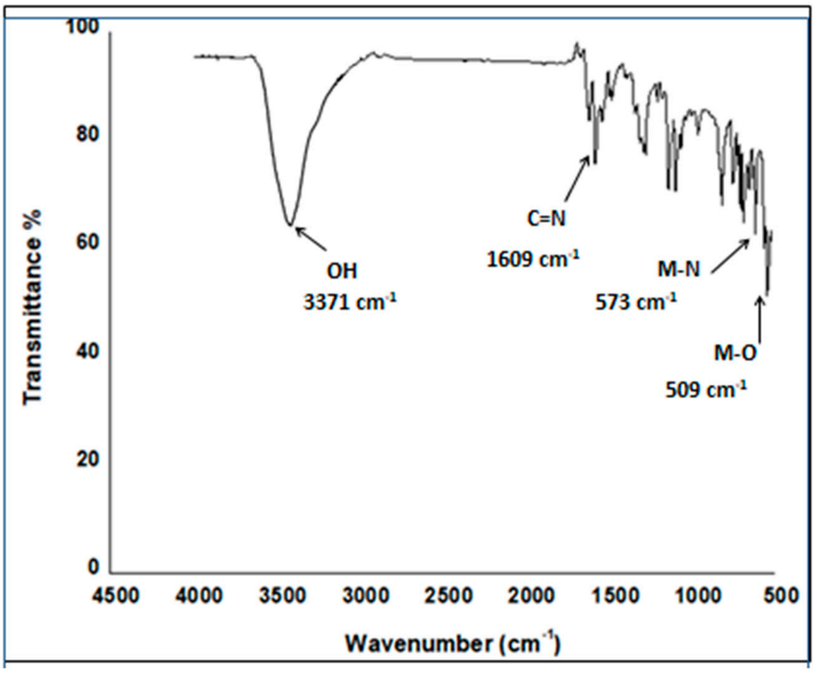

Figure S8: FT-IR spectrum of C_4.

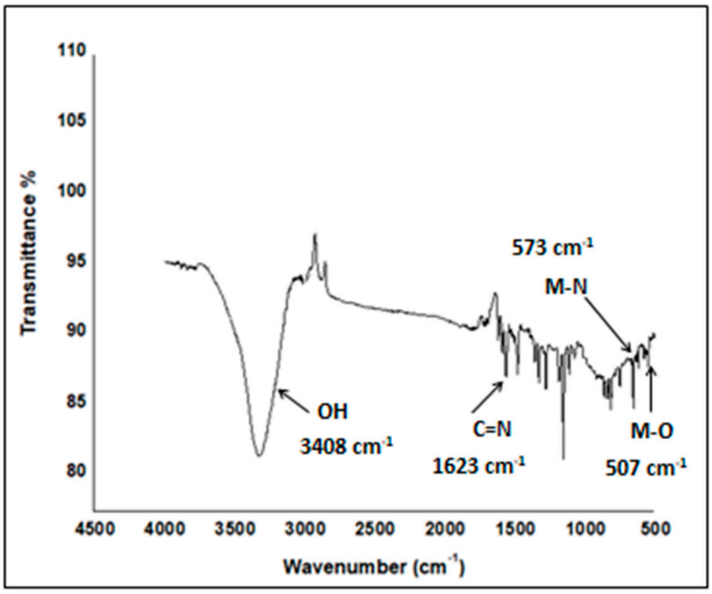

Figure S9: FT-IR spectrum of C_5.

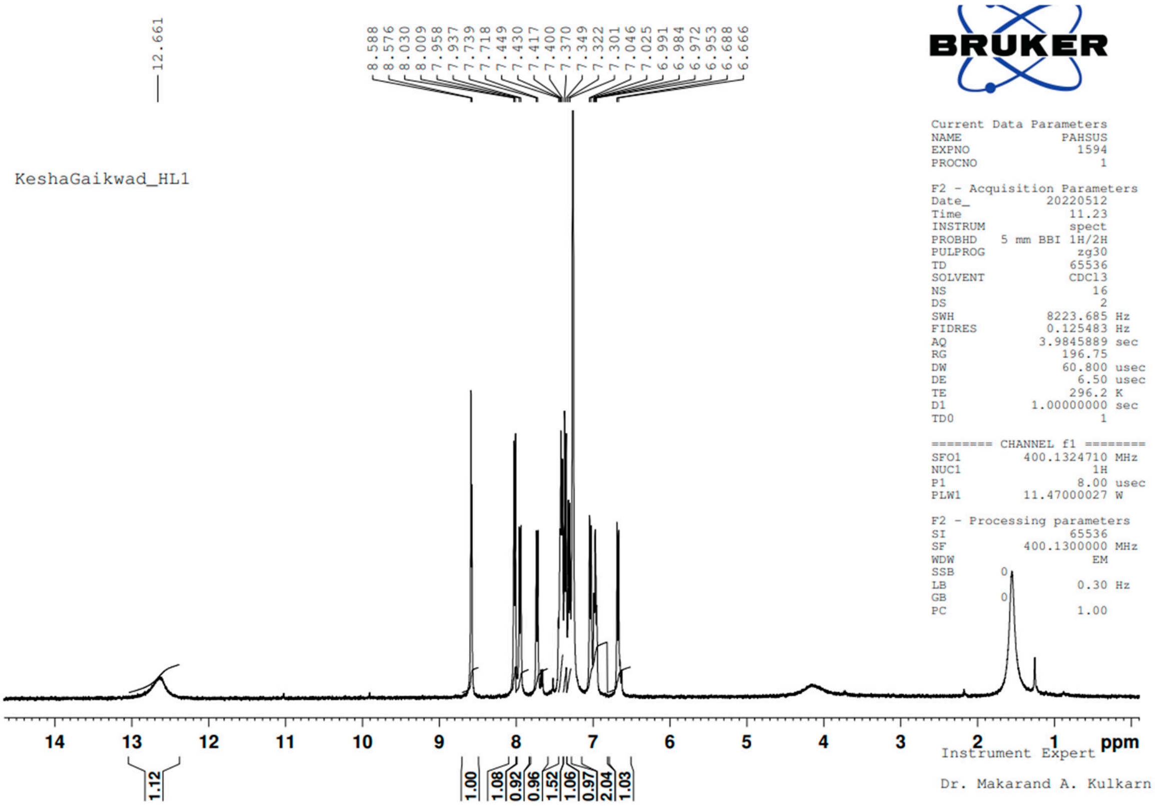

Figure S10: ^{1}H-NMR spectrum of **L**$_1$.

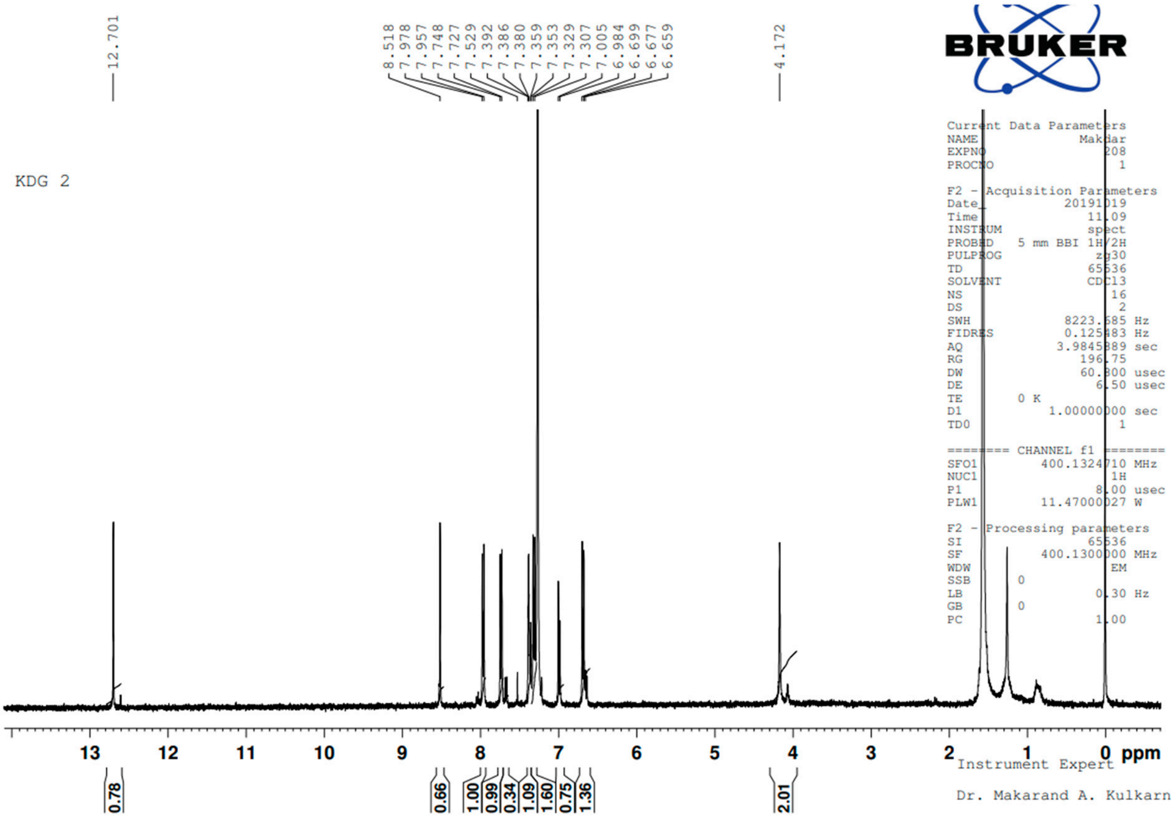

Figure S11: ^{1}H-NMR spectrum of **L**$_3$.

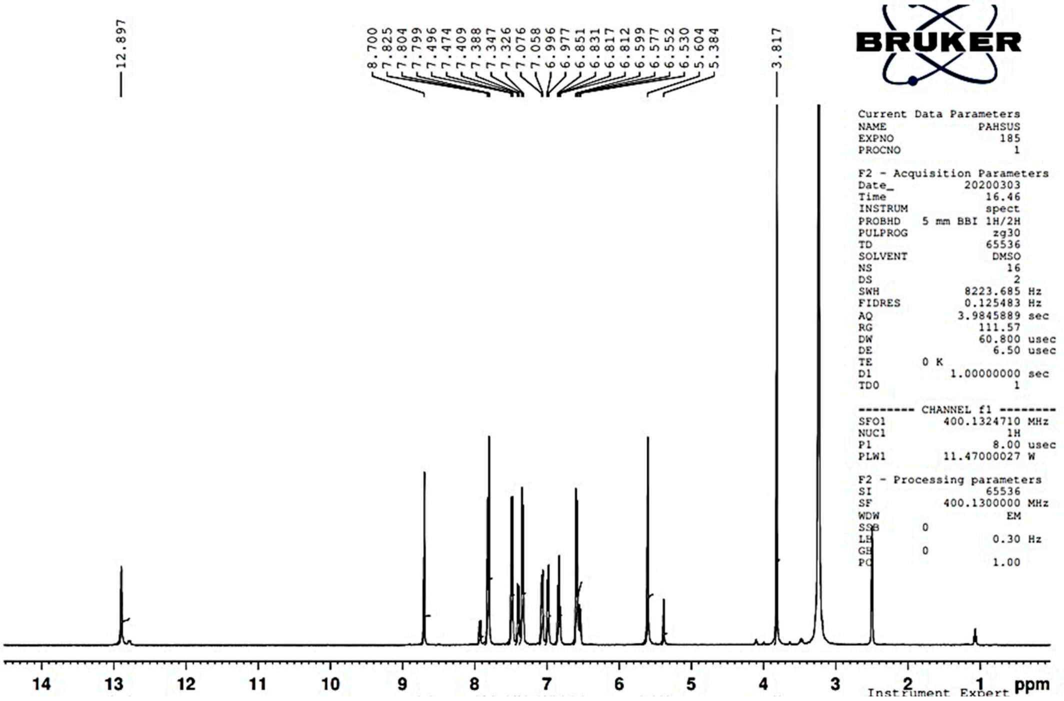

Figure S12: ^{1}H-NMR spectrum of **L$_4$**.

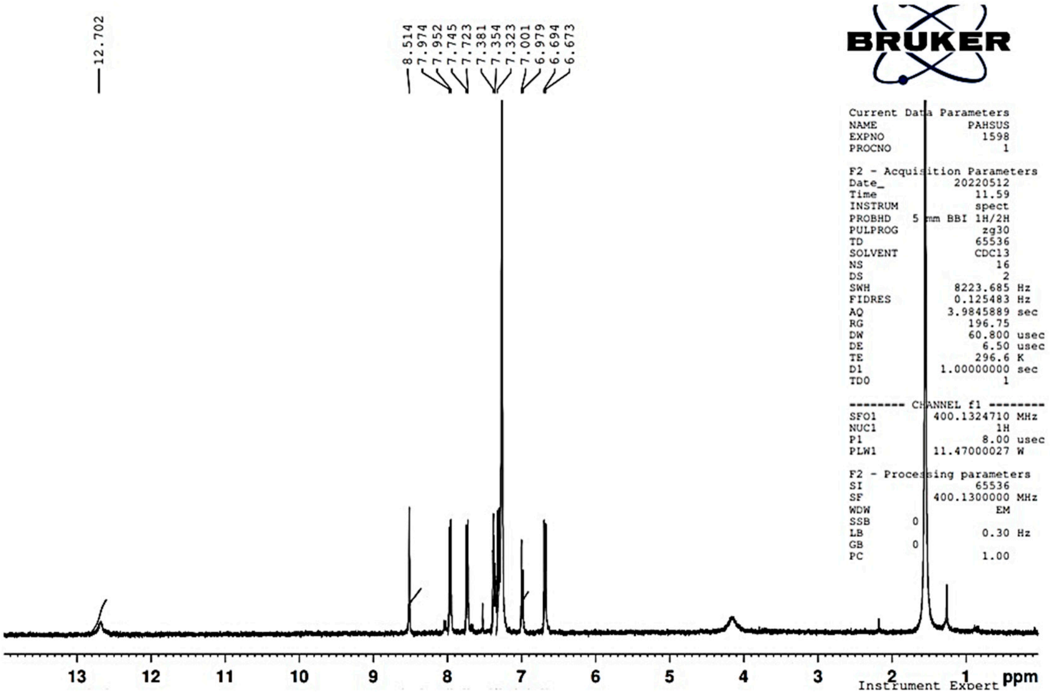

Figure S13: ^{1}HNMR spectrum of **L5**.

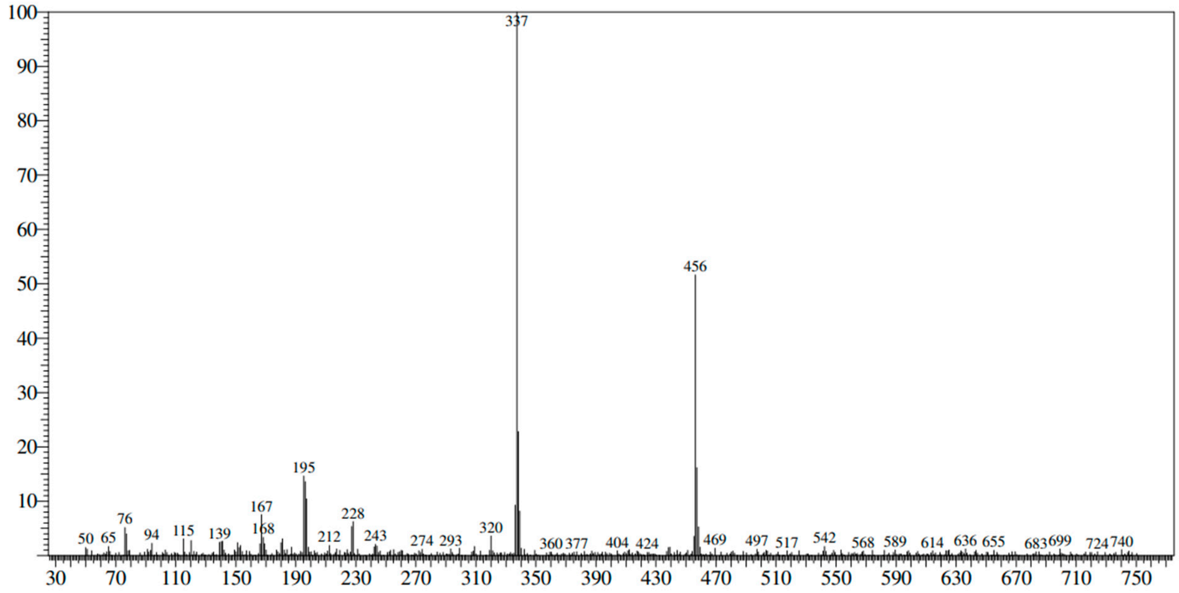

Figure S14: Mass spectrum of L_1.

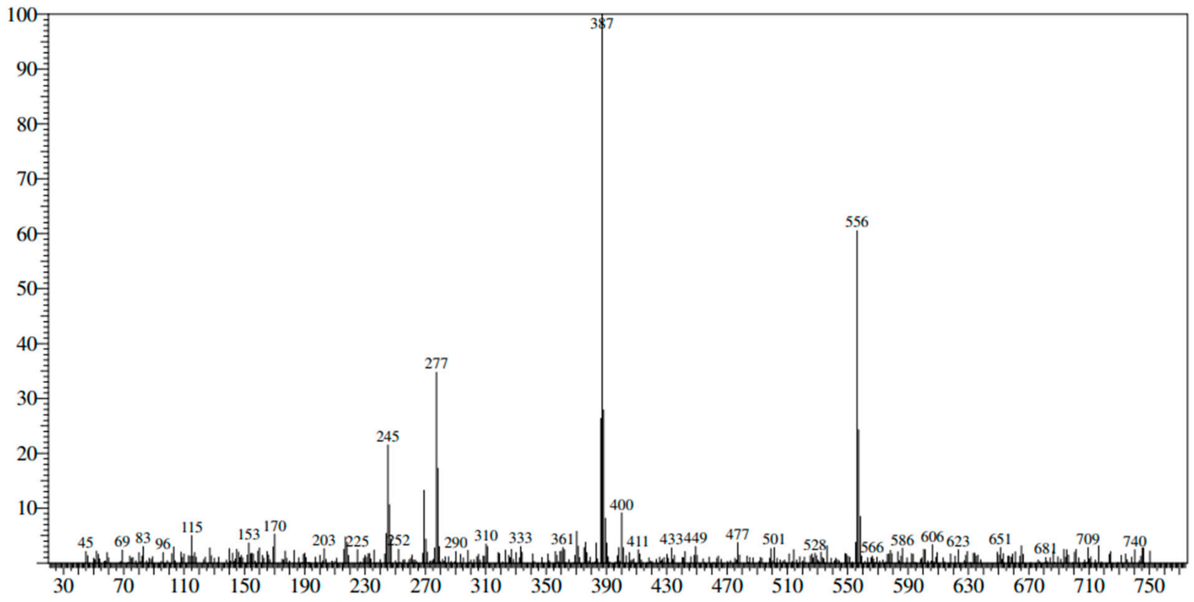

Figure S15: Mass spectrum of L2.

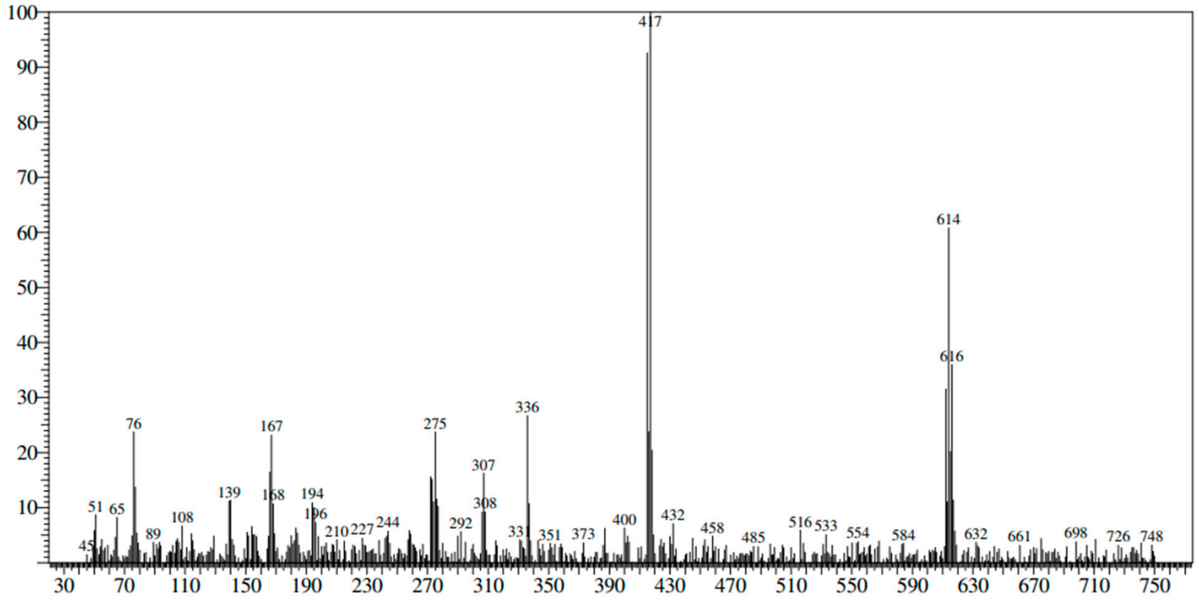

Figure S16: Mass spectrum of L_3.

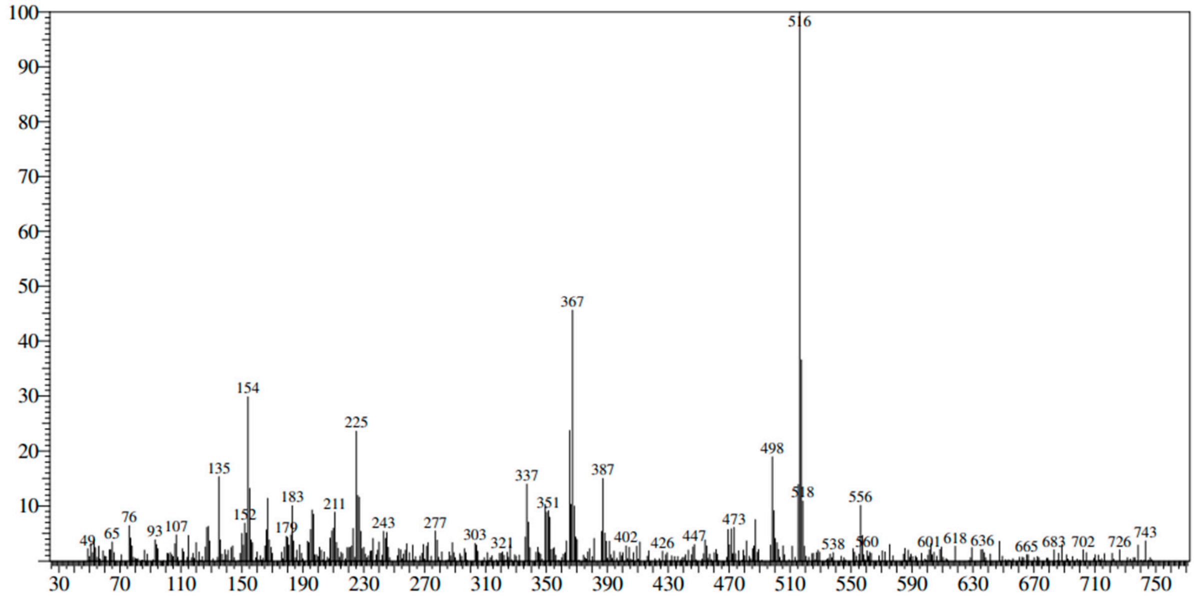

Figure S17: Mass spectrum of L_4.

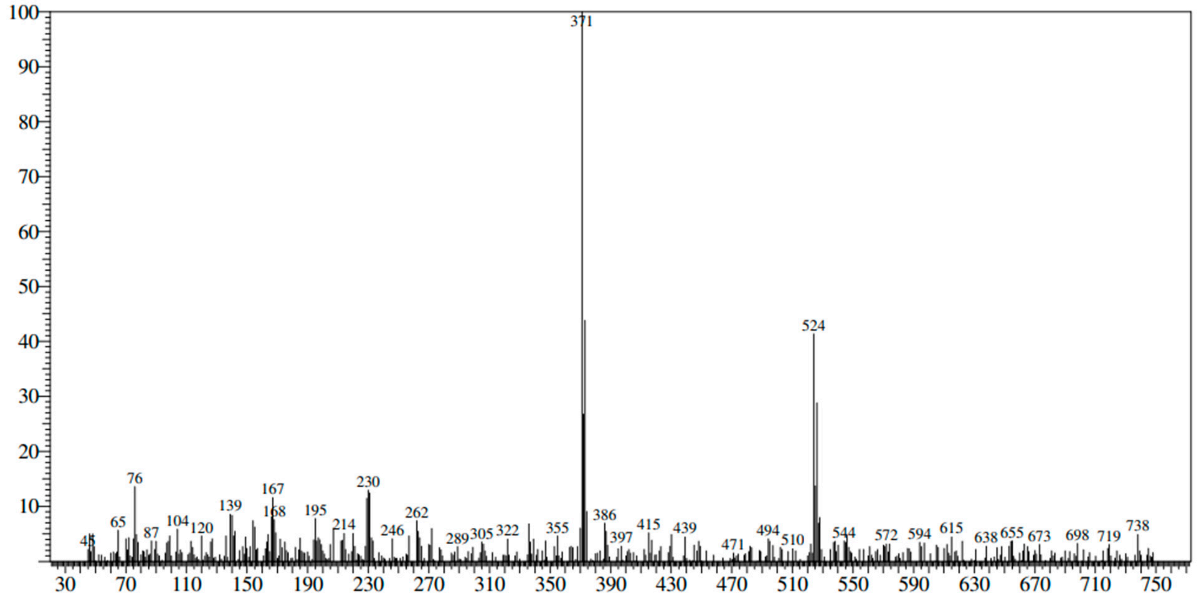

Figure S18. Mass spectrum of L5.

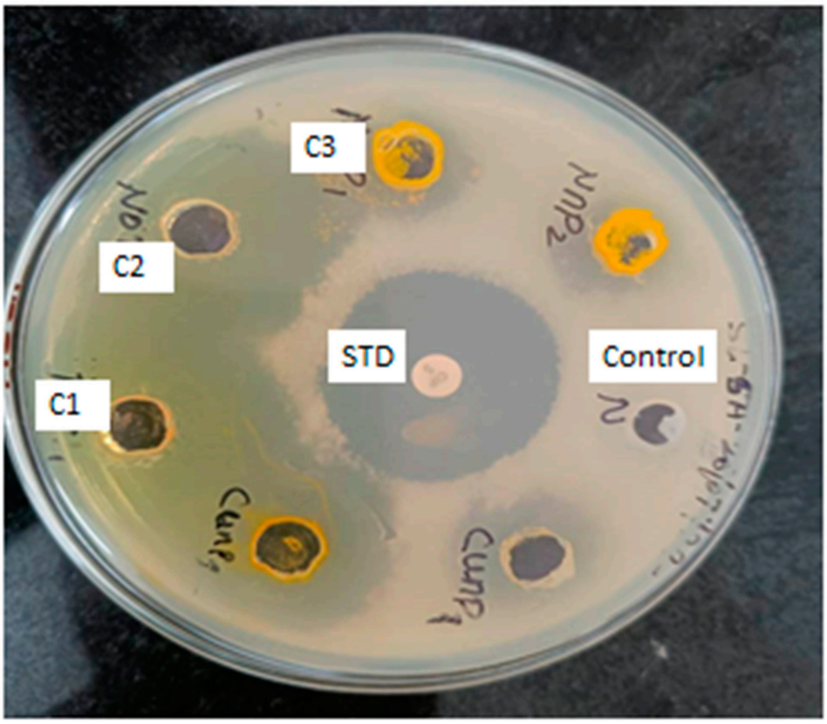

Figure S19: Antibacterial activity of Co(II) complexes and ligands against *Staphylococcus aureus.*

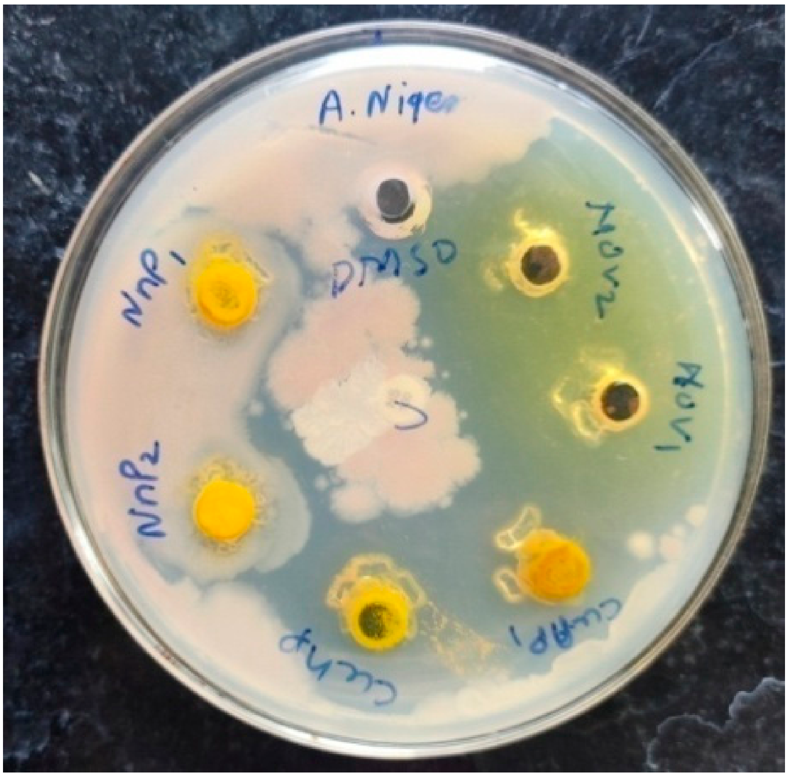

Figure S20: Antibacterial activity of Co(II) complexes and ligands against *Aspergillus niger.*

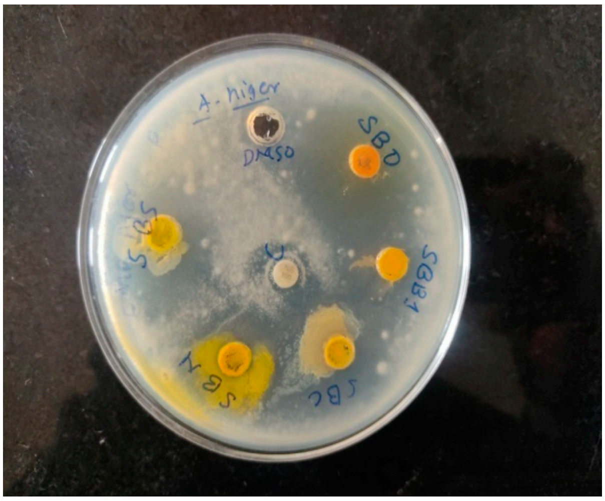

Figure S21: Ligand-Antibacterial activity against *Aspergillus niger.*

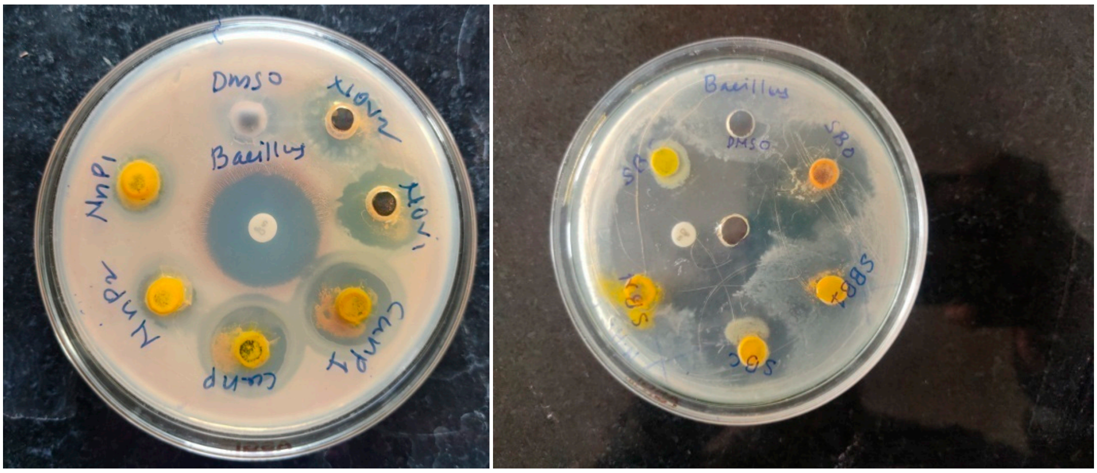

Figure S22: Antibacterial activity of Co(II) complexes and ligands against *Bacillus subtilis.*

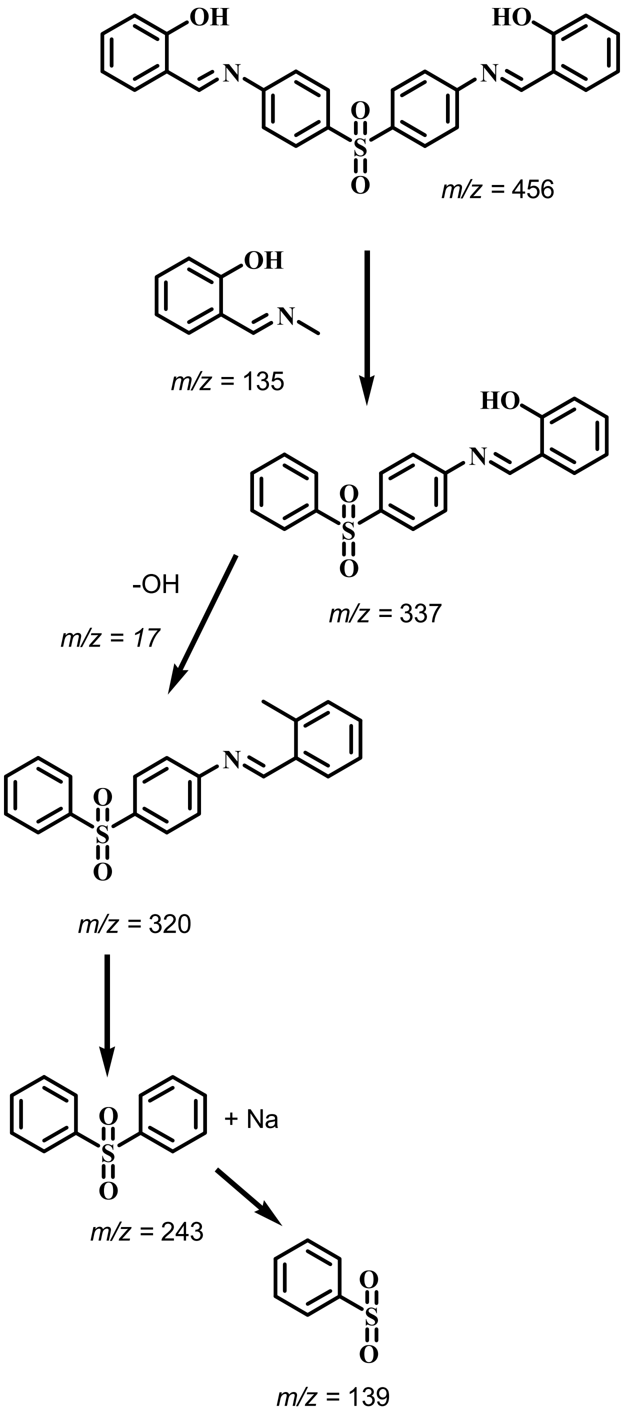

Figure S23: Mass fragmentation pattern of L_1.

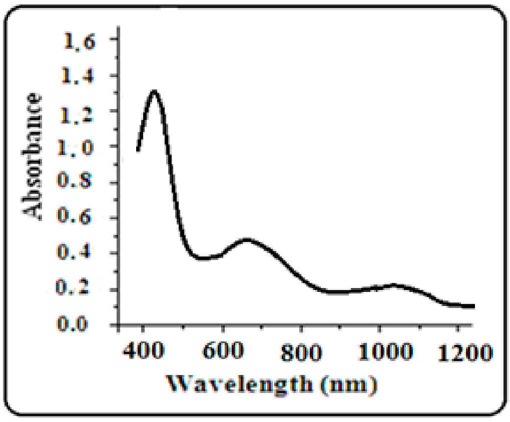

C1

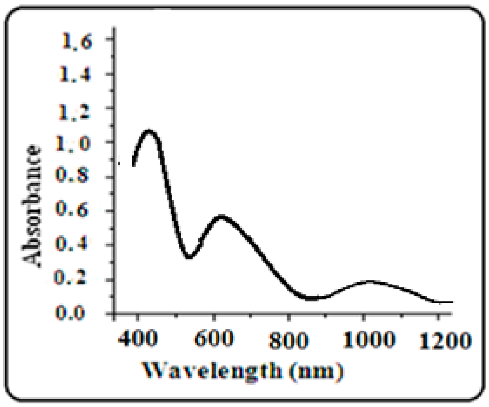

C3

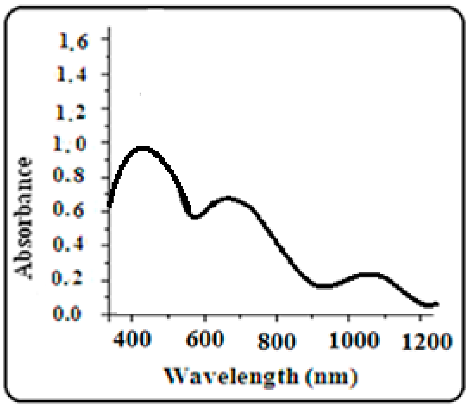

C5

Figure S24: UV-Visible absorption spectra of complexes.

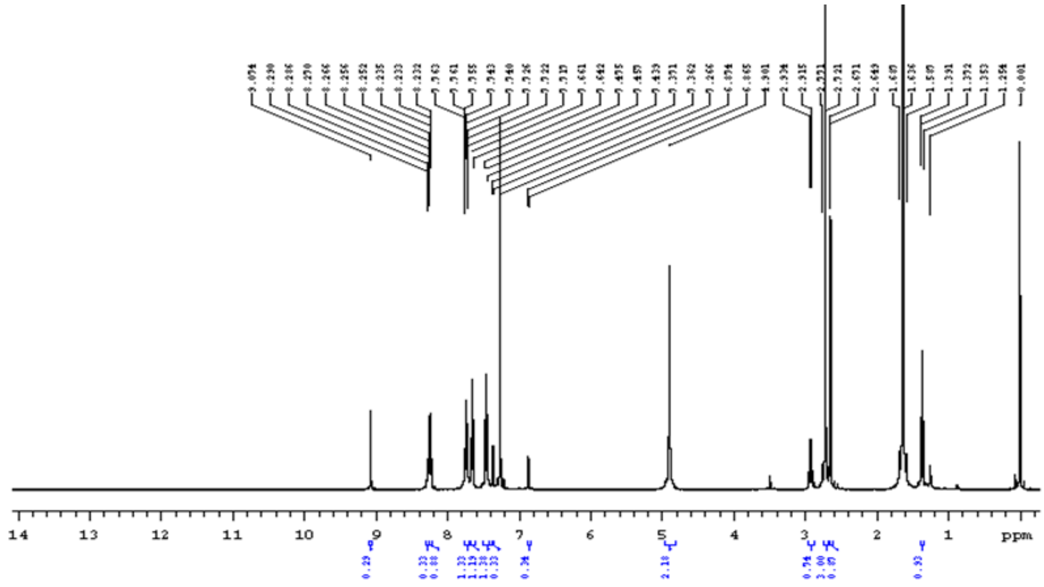

Figure S25: ^{1}H-NMR spectrum of C_1.

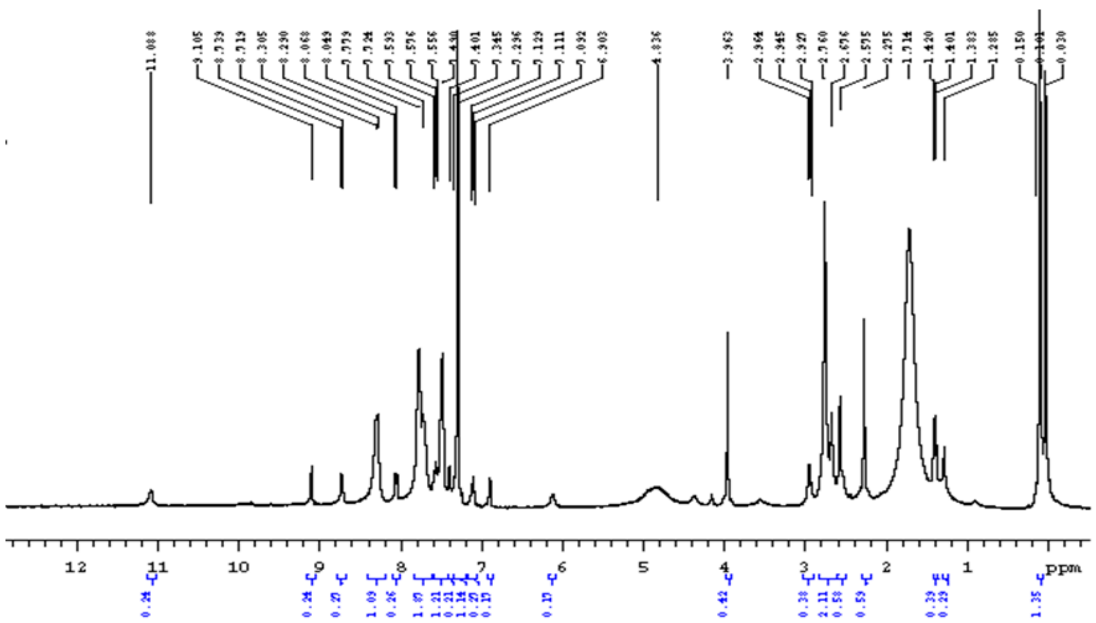

Figure S26: ^{1}H-NMR spectrum of C_2.

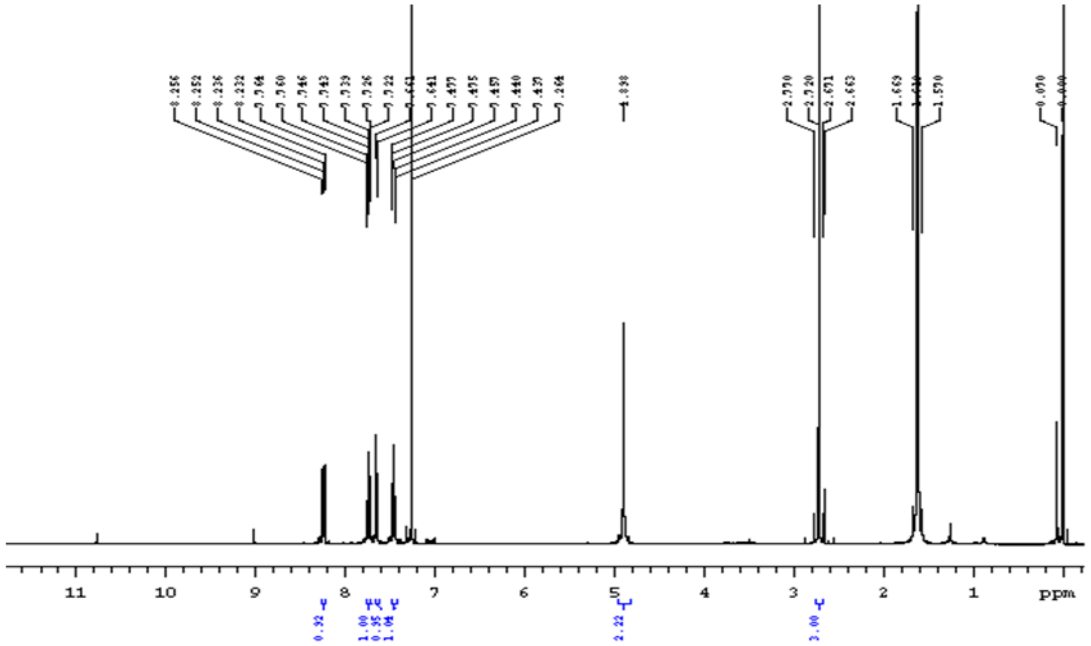

Figure S27: ^{1}H-NMR spectrum of C_3.

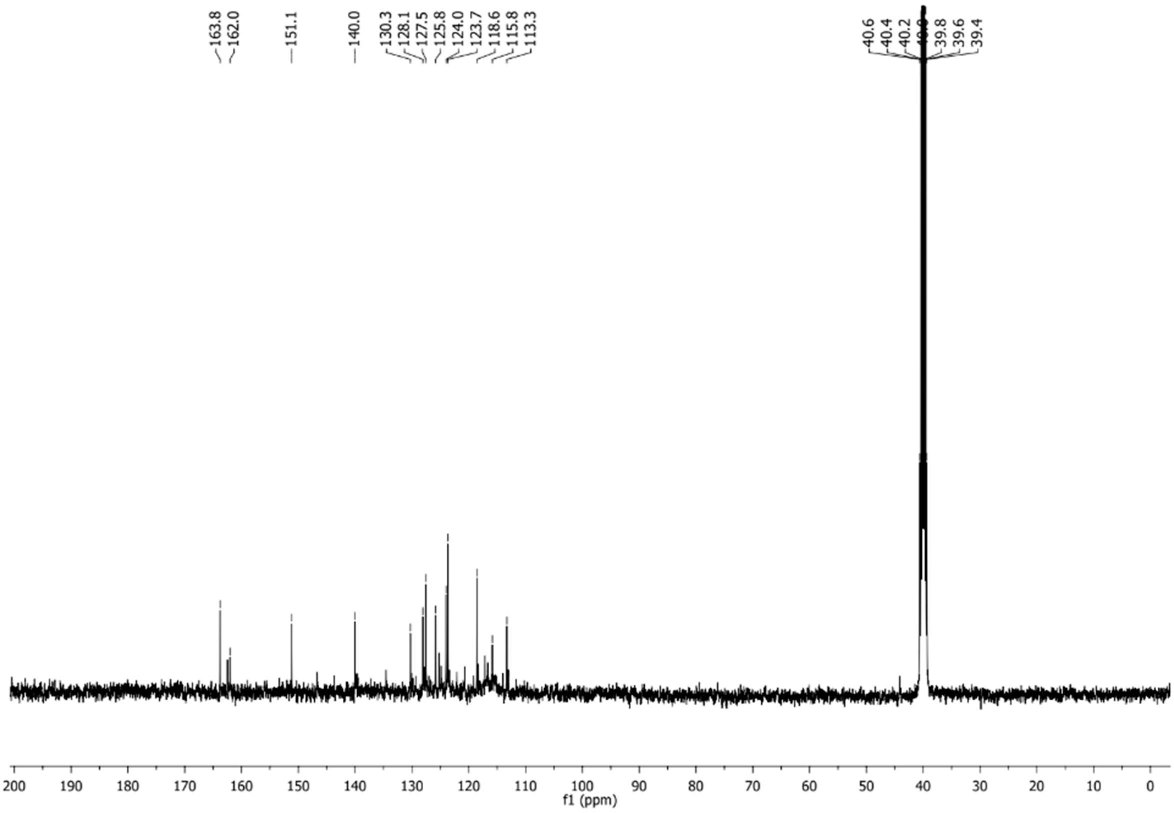

Figure S28: ^{13}C-NMR spectrum of C_1.

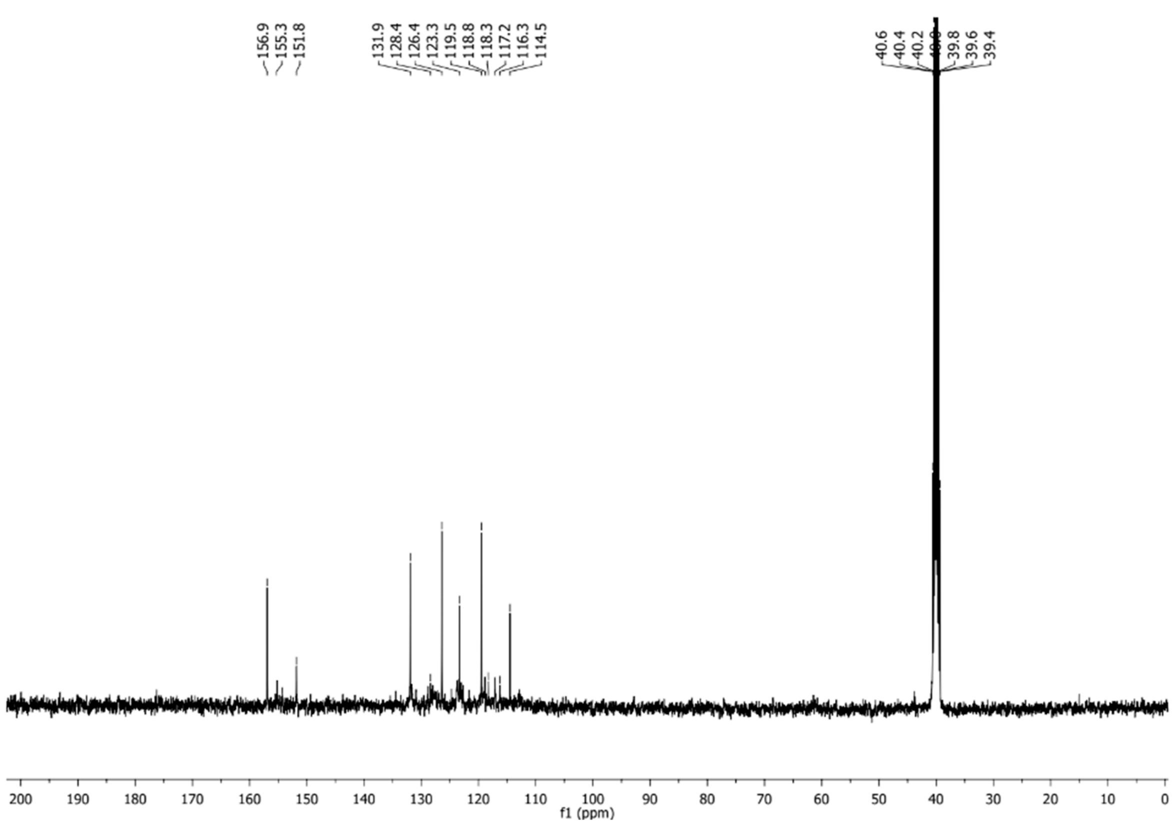

Figure S29: ^{13}C-NMR spectrum of C_2.

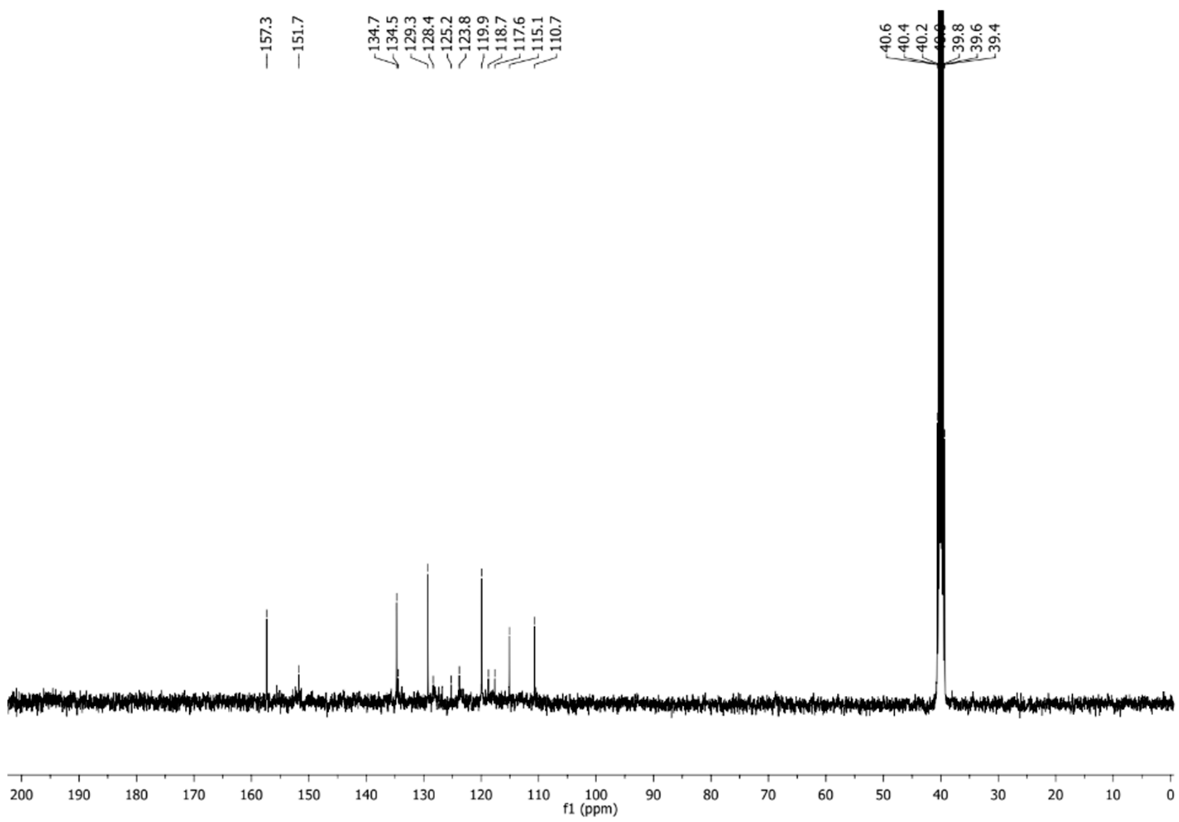

Figure S30: ^{13}C-NMR spectrum of C_3.
